# High Quantum Yield Amino Acid Carbon Quantum Dots with Unparalleled Refractive Index

**DOI:** 10.1021/acsnano.3c10792

**Published:** 2024-01-08

**Authors:** Vijay Bhooshan Kumar, Simcha K. Mirsky, Natan T. Shaked, Ehud Gazit

**Affiliations:** † The Shmunis School of Biomedicine and Cancer Research, George S. Wise Faculty of Life Sciences, Tel Aviv University, 6997801 Tel Aviv, Israel; ^‡^Department of Materials Science and Engineering and ^§^Department of Biomedical Engineering, Iby and Aladar Fleischman Faculty of Engineering, Tel Aviv University, Tel Aviv 6997801, Israel

**Keywords:** CQD, N-containing CQD, Amino acids, Refractive index, Quantum yield, Excitation-dependent emission, Live cell imaging

## Abstract

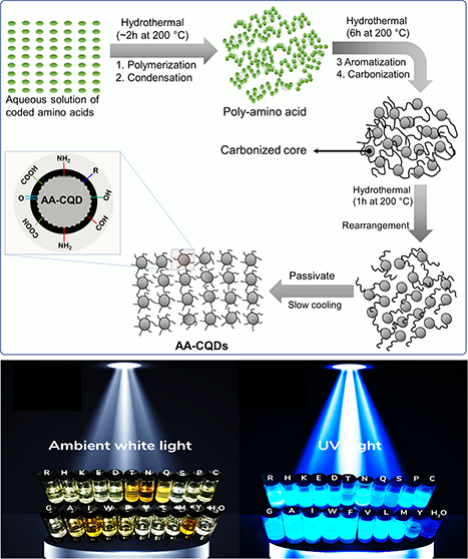

Carbon quantum dots (CQDs) are one of the most promising types of fluorescent nanomaterials due to their exceptional water solubility, excellent optical properties, biocompatibility, chemical inertness, excellent refractive index, and photostability. Nitrogen-containing CQDs, which include amino acid based CQDs, are especially attractive due to their high quantum yield, thermal stability, and potential biomedical applications. Recent studies have attempted to improve the preparation of amino acid based CQDs. However, the highest quantum yield obtained for these dots was only 44%. Furthermore, the refractive indices of amino acid derived CQDs were not determined. Here, we systematically explored the performance of CQDs prepared from all 20 coded amino acids using modified hydrothermal techniques allowing more passivation layers on the surface of the dots to optimize their performance. Intriguingly, we obtained the highest refractive indices ever reported for any CQDs. The values differed among the amino acids, with the highest refractive indices found for positively charged amino acids including arginine-CQDs (∼2.1), histidine-CQDs (∼2.0), and lysine-CQDs (∼1.8). Furthermore, the arginine-CQDs reported here showed a nearly 2-fold increase in the quantum yield (∼86%) and a longer decay time (∼8.0 ns) compared to previous reports. In addition, we also demonstrated that all amino acid based CQD materials displayed excitation-dependent emission profiles (from UV to visible) and were photostable, water-soluble, noncytotoxic, and excellent for high contrast live cell imaging or bioimaging. These results indicate that amino acid based CQD materials are high-refractive-index materials applicable for optoelectronic devices, bioimaging, biosensing, and studying cellular organelles *in vivo.* This extraordinary RI may be highly useful for exploring cellular elements with different densities.

## 1 Introduction

Carbon nanomaterials, including fullerenes, nanotubes, and graphene, have attracted considerable attention due to their exclusive physical, chemical, and biological properties.^1−3^ In addition to these highly important carbon nanomaterials, carbon quantum dots (CQDs) have emerged as an innovative class of fluorescent nanomaterials with a quasi-spherical morphology (less than 10 nm in all three dimensions) and distinctive physicochemical properties.^4,5^ As a result of the nanoscale dimensions, the mobility of electrons within the CQDs is restricted in all three directions. Due to their tunable photoluminescence (PL), chemical inertness, high water solubility, low fabrication cost, and low cytotoxicity, these materials can be used in a variety of applications.^6−9^ Several researchers have reported CQDs as a platform for drug delivery, gene delivery, cell imaging, and diagnosis utilizing fluorescence.^4^ A number of additional applications have been found for these materials, including metal sensing, photocatalysis, dye degradation, photosynthesis augmentation, and photovoltaics.^10^ Bright yellow CQDs have been synthesized with quantum yields up to 71% via an alkali-catalyzed molecular fusion strategy,^11^ red CQDs with graphitic carbon nitride have been synthesized using a facile coupling technique,^12^ and oxygen-doped carbon dots (O-CDs) that exhibit excellent performance as electrocatalysts^13^ have also been synthesized. Recently, Zhang et al. have also synthesized three types of graphene-based CQDs, which can be used to prevent single-layer formation and can directly interact with single-layer-containing protein.^14^ Furthermore, an EDC/NHS-assisted linking strategy was used to synthesize amide-based carbon quantum dots for heterojunction photocatalysis to enhance charge separation in heterojunction photocatalysts.^15^ CQDs can be synthesized from carbohydrates, proteins, lipids, and other biological molecules, as well as from renewable sources such as fruit and vegetable peels, nuts, and waste materials, using a bottom-up approach.^4^ Alternatively, using top-down methods, CQDs can also be synthesized from pure carbon of different forms, such as graphene, carbon nanotubes, coal, etc. In general, CQDs consisting of pure carbon or mainly carbon, oxygen, and hydrogen have low quantum yields (QYs) and fluorescence. To overcome this limitation and improve their fluorescence, N-doping is the most commonly used approach.^16^ In addition to the introduction of structural defects, this modification also introduces new energy states and increases the amount of electrons in the conduction band.^17^ In this regard, amino acids (AAs) are considered to be promising candidates for the preparation of CQDs with favorable optical properties as it is generally believed that the functionalization of CQD nanomaterials results in a homogeneous surface.^18^ It is important to note that AAs are renewable, abundant in nature, relatively inexpensive, and nontoxic.^19^ Yang et al. reported the formation of nitrogen-containing CQDs with blue luminescence by hydrothermal treatment of several AAs.^20^ Relative fluorescence analytical methods were used to determine a QY of 41.3%, the highest QY achieved by nitrogen-containing (N-containing) CQDs.^20^ Pandit et al. reported the formation of CQDs by pyrolyzing citric acid in the presence of various AAs under hydrothermal conditions.^21^ Kolanowska et al. have also attempted to fabricate CQDs by hydrothermal synthesis using only nine AA precursors.^22^ It should be noted, however, that no comprehensive study has been conducted on all CQDs based on all 20 coded AA for physicochemical or biological purposes. Therefore, it is necessary to demonstrate how AA-derived CQD fluorescence can be influenced in terms of the quantum yield, decay time, biological activity, and refractive index (RI).

RI is defined as the ratio of the speed of light in a vacuum to the speed of light in a material and is dependent on the wavelength.^23^ Light scattering is related to the size, shape, and chemical composition of nanomaterials,^24^ and it defines the optical force exerted on nanomaterials by electromagnetic fields.^25,26^ The RI determines how light interacts with nanomaterials^27^ in a variety of applications such as pharmaceuticals,^28^ drug delivery,^29^ biosensors,^30^ nanomedicine therapy,^31^ and materials science.^32−34^ Additionally, it is necessary to use high-RI materials when designing optical fibers and optical devices, such as optical interference filters and mirrors, optical sensors, solar cells, eyeglass lenses, and biomedical devices, in order to optimize their performance. A number of polymers, polymer composites, and carbon materials with high RIs have attracted significant interest in recent years due to their lightweight properties and excellent flexibility and formability in comparison to inorganic materials.^35^ The initial report describing the measurement of the RI of colloid carbon was published by Janzen et al. in 1975 using paracrystalline colloidal carbons.^36^ Subsequently, carbon RI has been assessed based on extinction measurements performed on a specially-made carbon black containing very small particles at visible wavelengths (350–1000 nm).^36^ Furthermore, nanographene’s plasmon-enhanced optical response over the visible and near-infrared spectrum was shown to provide an ideal platform for the development of electrically tunable nonlinear optical nanodevices.^37^ Their optical properties made them particularly appealing as opto-functional materials, which led to the observation of different physical phenomena, such as RI changes.^38^ It was further reported that the RIs of individual carbon materials are affected by photon energy using a nonlinear optical device.^39^ It was also reported that CQDs incorporated into polyvinyl alcohol (PVA) nanocomposites show a maximal RI of 1.84.^40^ Nevertheless, the RIs of all CQDs based on each of the different AAs are unknown. In order to facilitate the design of CQDs, it is highly desirable to obtain a comprehensive understanding of their RI and surface functionality as a function of the AA concentration.

The main focus of this research article is the systematic exploration and optimization of the formation of carbon quantum dots (CQDs) from all 20 coded AAs. Our study involved the use of 20 different AAs as precursors for the synthesis of 20 different CQDs from 20 coded AAs in an aqueous solution. An overview of the chemical structures of all AAs is provided in Figure S2 in the Supporting Information. All 20 coded AA-CQDs possess different functional groups such as −COOH, −NH_2_, −OH, C=O, −COOR, and −NHR offering a variety of chemical reactions. In this study, we describe a facile and sustainable one-step hydrothermal method for synthesizing CQDs from all 20 coded AAs. Four-stage synthesis was carried out in a fully controlled manner, namely, dehydration, polymerization, passivation, and carbonization. We demonstrate that it is possible to obtain CQDs from all 20 coded AAs showing a wide-range fluorescence in the visible spectrum and exhibiting red shifts depending on the synthetic precursors of the AAs. The CQDs derived from single AAs show high RIs, high QYs, narrow size distributions, and excellent water solubility. Additionally, we propose a synthesis technique for AA-derived CQDs (AA-CQDs), where AAs are functionalized with CQD nanomaterials, resulting in RI, decay time, and QY that can be varied and reach values of up to 2.15, 8.6 ns, and 86%, respectively. We achieved CQDs with the highest RIs ever reported for any type of CQD. Moreover, the CQDs exhibit low toxicity and high biocompatibility, high PL intensity, high chemical stability, and excellent biological, physical, and chemical properties, making them suitable for a wide range of applications.

## 2 Results and Discussion

### 2.1 Synthesis of AA-CQD Nanomaterials from AAs

A one-pot facile method consisting of two chemical steps was utilized for the synthesis of AA-CQDs. As a first step, polymerization and carbonization processes were performed to form the core, while the second step involved the conjugation of AAs under hydrothermal conditions in an autoclave to control the carbonization process.

### 2.2 Physicochemical Characterizations

Visual analysis, fluorescence, UV–vis absorption, fluorescence decay time, and transmission electron microscopy (TEM) studies were carried out to confirm that CQDs were formed from the aqueous solution of AA via a hydrothermal reactor. Visual inspection of aqueous suspension of CQDs synthesized from AAs and aqueous suspension of pristine AAs was performed in ambient white light and UV light (Figure S1; see the Supporting Information). Light pale-yellow to dark pale-yellow were the predominant colors of the synthesized CQDs in ambient white light (Figure S1a), while the corresponding aqueous solutions of pure AAs (all AA chemical structures have been drawn in Figure S2) were predominantly transparent and colorless (Figure S3a). Under UV light (365 nm), the same CQD materials exhibited a blue-white to green-white fluorescence (Figure S1b), while the aqueous pure AA solutions displayed no fluorescence under the same UV light (Figure S3b). Even after a long and continuous exposure to white light or UV light, the fluorescence of the solutions did not change.

CDs are commonly constructed of a carbon core and an amorphous shell with oxygen-containing groups. AAs were used as the carbon and nitrogen source for CQD synthesis and also provided surface passivating groups such as −NH_2_, COH, −COOH, −C=O and −OH, thereby generating a carbon core bond with nitrogen and an amorphous shell with amino groups and oxygen-containing groups. According to the formation mechanism of CDs proposed by Kumar et al.,^41^ CQDs synthesized from all coded AAs undergo four stages, including dehydration, polymerization, carbonization, and surface passivation, as depicted in Figure S1c. Examining His-CQDs as an example, the formation process of AA-CQDs via hydrothermal treatment at 200 °C is as follows. In the primary stage of hydrothermal treatment, small AA monomers undergo dehydration and polycondensation reactions between carboxyl and amino groups, as has been shown by Kumar et al.^41,42^ and other researchers.^18^ In contrast to the previous study, our proposed mechanism is a temperature- and time-dependent hydrothermal reaction. When the solution temperature reached roughly 200 °C, AA amide and hydronium ions were formed via a reaction between two fragments of the AA molecules. Further heating initiated condensation of the poly-AA by dehydration and dehydrogenation of AA molecules. A short hydrothermal treatment (2–3 h) led to the polymerization of AA and the formation of AA-CQDs. In addition, after 6–10 h of hydrothermal treatment at 200 °C, the polymers began to carbonize, and subsequently, large-sized particles began to shrink via a continuous carbonization process, leading to a short single burst of nucleation. With a prolongation of the hydrothermal time, very uniform and monodispersed AA-CQDs could be obtained. In the process of hydrothermal treatment, N elements were doped into the carbon framework to form an N-doped CQD core with graphene-like carbon ring structures, and the N-doping amount increased with prolonged hydrothermal reaction time.^43−45^ In addition, the CQDs core was peripherally modified with amino groups and oxygen-containing groups to form a shell of surface passivation. As amino groups and oxygen-containing groups were unstable and tended to leave the surface after a longer hydrothermal reaction time, the surface passivation was a time-dependent process. We have observed that aqueous dispersions of CQDs were transparent and slightly yellow to dark yellow under white light and remained stable for up to several weeks. Upon excitation under a 345 nm UV lamp (Figure S1b), the CQDs emitted a strong white-blue to green-blue luminescence and the QY reached up to 86% for certain AA-CQDs such as Arg-CQDs and His-CQDs.

**Figure 1:**
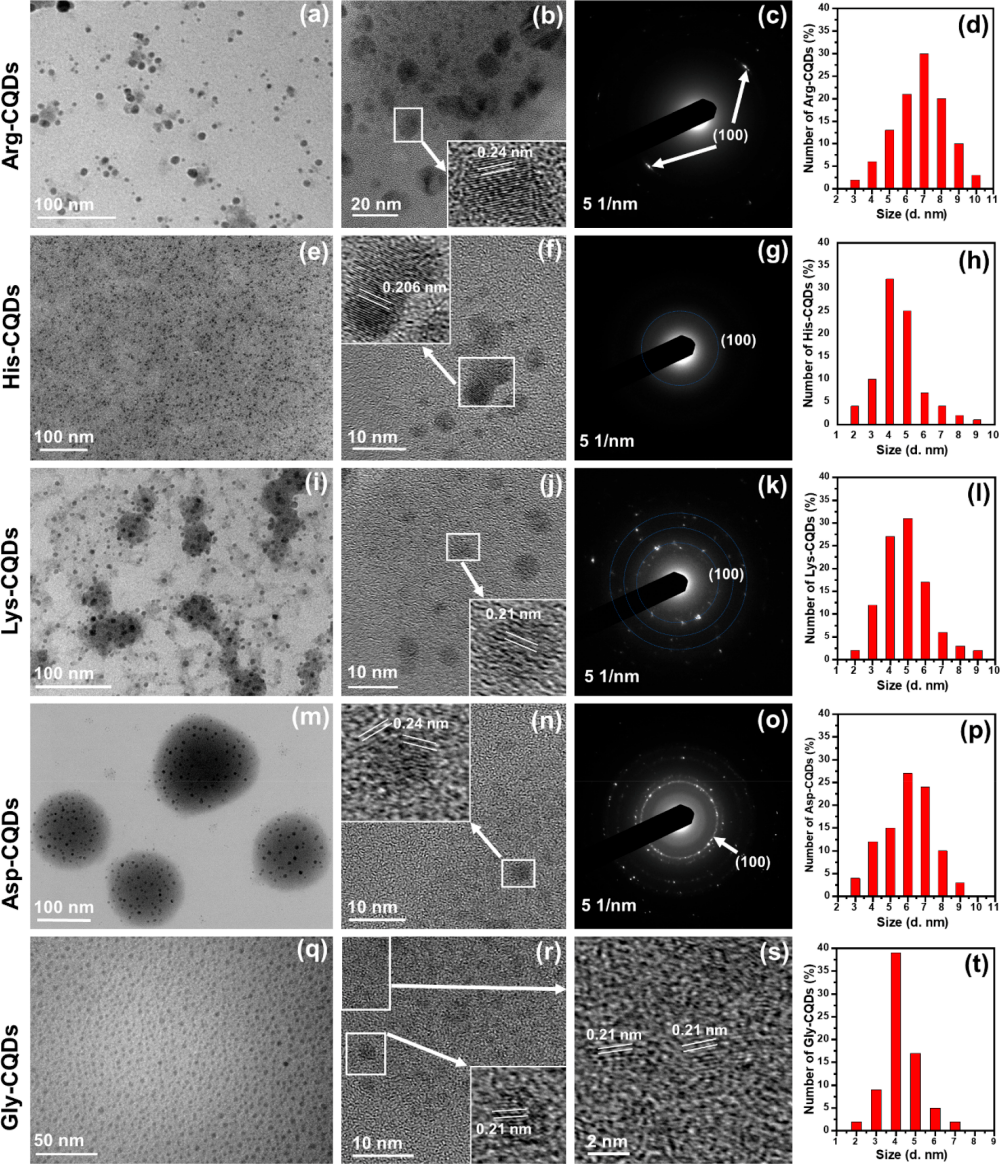
Morphological analysis of AA-CQDs using TEM, HRTEM, and SAED: (a–d) Arg-CQDs, (e–h) His-CQDs, (i–l) Lys-CQDs, (m–p) Asp-CQDs, and (q–t) Gly-CQDs. (a, e, i, m, q) TEM micrographs, (b, f, j, n, r) HRTEM images (with magnified inset to show lattice fringes), (c, g, k, o) SAED patterns, and (d, h, l, p, t) particle size histograms. (s) A magnified portion of (r).

**Figure 2:**
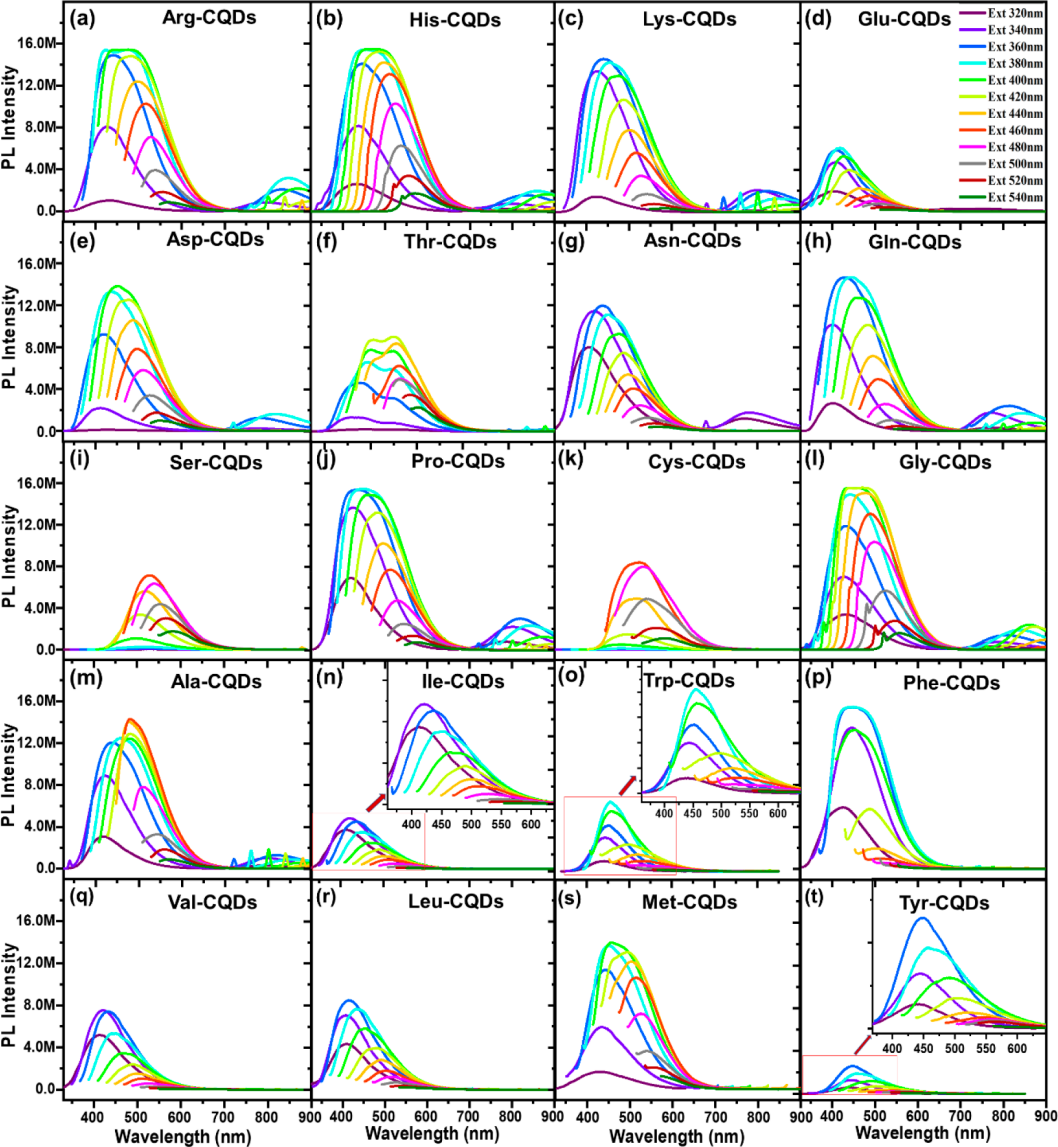
Fluorescence spectra of the AA-CQDs: (a) Arg-CQDs, (b) His-CQDs, (c) Lys-CQDs, (d) Glu-CQDs, (e) Asp-CQDs, (f) Thr-CQDs, (g) Asn-CQDs, (h) Gln-CQDs, (i) Ser-CQDs, (j) Pro-CQDs, (k) Cys-CQDs, (l) Gly-CQDs, (m) Ala-CQDs, (n) Ile-CQDs (inset: magnified portion of the fluorescence spectrum of Ile-CQDs), (o) Trp-CQDs (inset: magnified portion of the fluorescence spectrum of Trp-CQDs), (p) Phe-CQDs, (q) Val-CQDs, (r) Leu-CQDs, (s) Met-CQDs, and (t) Tyr-CQDs (inset: magnified portion of the fluorescence spectrum of Tyr-CQDs).

The morphologies of AA-CQDs synthesized under hydrothermal conditions were examined by using TEM and high-resolution TEM (HRTEM) (Figure 1). CQD diameters ranging from 3.0 ± 0.5 nm to 9.0 ± 0.8 nm were observed, with size depending on the AA used as a precursor. The TEM images for Arg-CQDs show uniform spherical particles (Figure 1a and Figure S4). Based on Figure S4, the particle size distribution was calculated, and a histogram of the particle size distribution is shown in Figure 1d, with an average value of 6.0 ± 0.5 nm. HRTEM analysis (Figure 1b) showed that the Arg-CQDs had clear lattice fringes, with 0.21 nm *d* spacing, indicating that these materials are highly crystalline. In addition, we studied the selected area electron diffraction (SAED) pattern and found it to display the ring pattern of graphitic diffraction of an sp^2^ structure with (100) planes.^46^ Based on the TEM analysis (Figure 1e and Figure S5), it appears that the His-CQDs were mostly spherical and were generally well-dispersed. HRTEM images and SAED patterns of His-CQD nanomaterial samples are shown in Figures 1f,g, demonstrating the graphitic nature of carbon with a 0.206 nm *d* spacing and (100) planes. Based on Figure S5, we plotted the particle size distribution and found that the average particle size was 4.0 nm (Figure 1h). TEM images of Lys-CQDs are shown in Figures 1i,j and Figure S6. These nanomaterials were uniform and monodispersed with a near-spherical shape and an interlayer spacing of approximately 0.21 nm (inset of Figure 1j). This is indicative of the formation of graphite structures, as Figure 1k was close to the (100) peak of graphite.^41^ According to a statistical histogram of Lys-CQD sizes, the average size was 5 nm (Figure 1l), ranging from 3 to 8 nm. TEM analysis of the Asp-CQDs (Figure 1m and Figure S7) and Gly-CQDs (Figure 1q and Figure S8) showed uniform and monodispersed nanomaterials with near-spherical shapes. HRTEM images of the Asp-CQDs (Figure 1n) and Gly-CQDs (Figure 1r,s) revealed their crystalline nature with an interlayer spacing of ∼0.24 nm (inset in Figure 2n) and 0.21 nm (inset in Figure 1r, and Figure 1s), respectively, indicating that graphite structures had been formed. Moreover, we have observed that several hundreds of Asp-CQDs are surrounded by a few larger (100 nm) drop-like structures, which are not present in high-resolution TEM, indicative of pseudostructures produced by the solvent. In addition, a statistical histogram showed a narrow range of sizes, ranging between 3 and 9 nm (Asp-CQDs, Figure 1p and Figue S7) and between 3 and 7 nm (Gly-CQDs, Figure 1t and Figure S8). A purity analysis of one of the CQDs was conducted by using energy-dispersive X-ray spectroscopy (EDS) associated with HRTEM analysis (Figure 1f). His-CQDs were found to contain C, N, and O (Figure S9), indicating that the sample did not contain any impurities. The morphology of all the remaining AA-CQDs was similarly analyzed by TEM electron microscopy; see Figures S10–S24 for Glu-CQDs, Thr-CQDs, Asn-CQDs, Gln-CQDs Ser-CQDs, Pro-CQDs, Cys-CQDs, Ala-CQDs, Ile-CQDs, Trp-CQDs, Phe-CQDs, Val-CQDs, Leu-CQDs, Met-CQDs, and Tyr-CQDs, respectively. Table 1 provides a summary of the average particle sizes of the AA-CQDs.

**Table 1:**
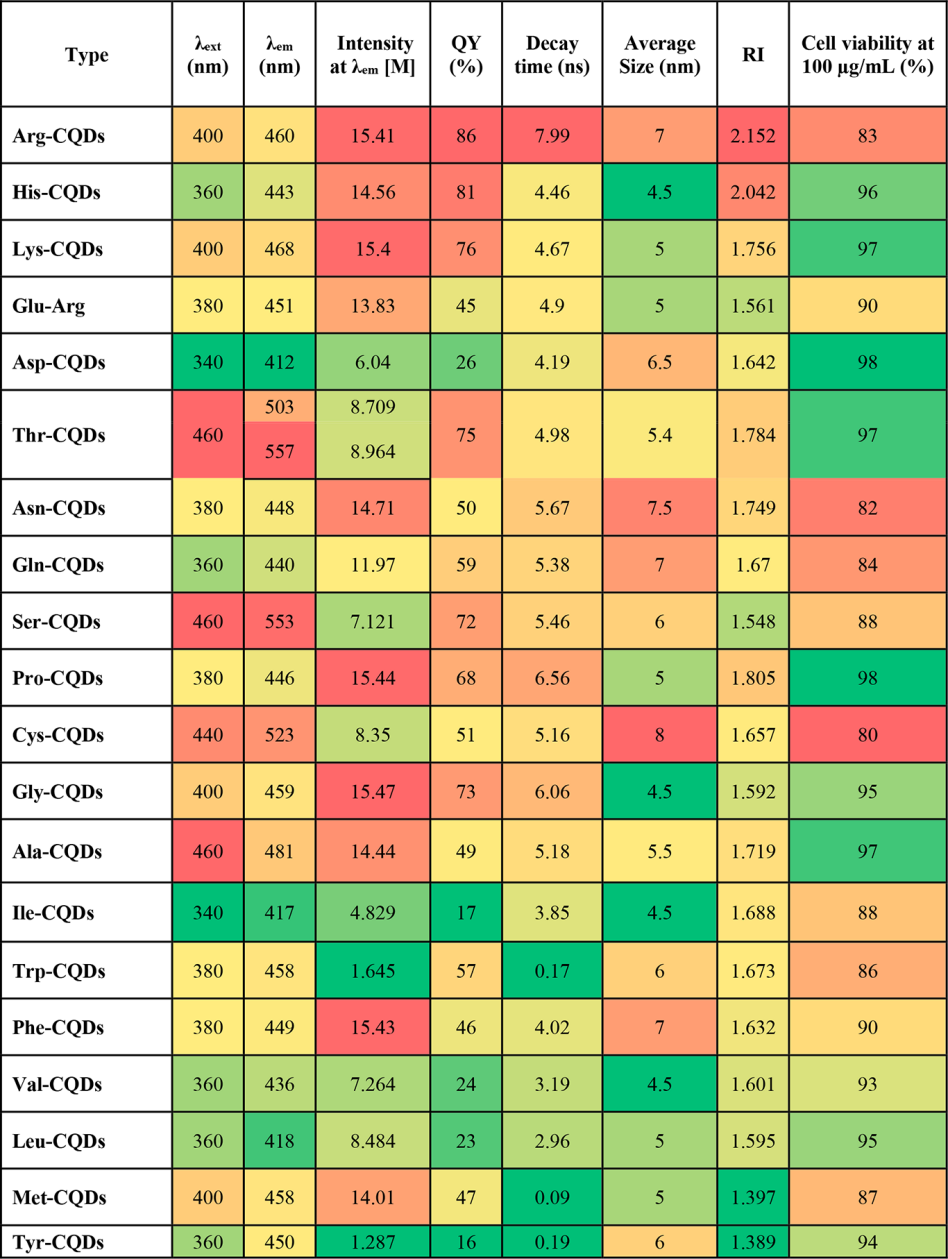
Measured Properties of All AA-CQD Nanomaterials

Various spectroscopy methods were used to analyze the synthesized AA-CQDs, including UV–vis, fluorescence, and X-ray photoelectron spectroscopy (XPS) and EDS. UV–vis spectroscopy revealed different characteristics of AA-CQD absorption peaks. Three distinct absorption peaks were observed in the UV–vis spectra of the AA-CQDs (Figure S25), suggesting that the AAs were efficiently conjugated to the surface of the CQDs and formed long-chain polyamides and polyesters.^47^ This long-chain residue assembled and provided a supramolecular, polymeric, and compact structure, which exhibited a sharp high-energy absorption peak at 235 nm and a broad low-energy absorption peak around 340 nm due to the electronic transition from π to π* of the sp^2^-hybridized carbon–heteroatom bond (C=O and C=N). In addition, the absorption band around 260–270 nm corresponds to the AAs. The absorbances of coded AA-derived CQDs at 240 and 340 nm are associated with some of the self-assembled AAs on the surface of CQDs. The absorption peaks at 270 and 360 nm were detected in the absorption spectra of all AA-CQDs with high-energy (π–π*) and low-energy (n-π*) electron transitions, respectively (Figure S25). The UV–vis absorption spectra show that the supramolecular, compact, polymeric, assembled molecular structure attached to AAs exhibits tunable optical properties.

Previously, the excitation-dependent fluorescence property of CQDs has been studied by doping various elements and functionalizing surfaces with different functional groups.^4,40,48^ However, to our knowledge there have been no reports regarding the modification of surfaces by CQDs derived from all 20 AAs. We studied the effect of each of the 20 AA residues in diversely functionalized AA-CQDs at different excitation wavelengths (320–540 nm) and observed excitation-dependent emission in the range of 440–740 nm. Molecular fluorophores produced excitation-dependent emission that remained on the surface of CQDs at both the core and the defect sites. Specifically, we observed the highest fluorescence for Arg-CQDs (Figure 2a), His-CQDs (Figure 2b), Lys-CQDs (Figure 2c), Asp-CQDs (Figure -2e), Asn-CQDs (Figure 2g), Gly-CQDs (Figure 2i), Pro-CQDs (Figure 2j), and Met-CQDs (Figure 2s). In particular, excitation at 340–360 nm produced a highly intense emission spectrum at 400–450 nm for His-CQDs (Figure 2b), Asp-CQDs **(**Figure 2e), Gln-CQDs (Figure 2d), Ile-CQDs (Figure 2n), Val-CQDs (Figure 2q), Leu-CQDs (Figure 2r), and Tyr-CQDs (Figure 2t). Additionally, upon excitation between 440 and 480 nm, high emissions between 500 and 800 nm were observed for CQDs such as Thr-CQDs, Ser-CQDs, Cys-CQDs, and Ala-CQDs (Figure 2f–j). The Thr-derived CQDs exhibited a different fluorescence pattern compared to the other 19 AA-CQDs, with two emission peaks at 505 and 560 nm instead of one emission peak at 460 nm. The two emission peaks may also represent two photonic states of the material. Low fluorescence was observed for Ile-CQDs (Figure 2n), Val-CQDs (Figure 2q), Leu-CQDs (Figure 2r), Tyr-CQDs (Figure 2t), and Trp-CQDs (Figure 2o). The excitation and emission values are listed in Table 1. As a control, the fluorescence of pristine AA molecules in aqueous solutions was measured, showing that AA-CQD nanomaterials exhibited extremely high fluorescence compared to pristine AA molecules. Comparative fluorescence analysis graphs of all the AA-CQDs and the corresponding aqueous solutions of pristine AAs are shown in Figure S26.

### 2.3 Chemical Characterizations

**Figure 3:**
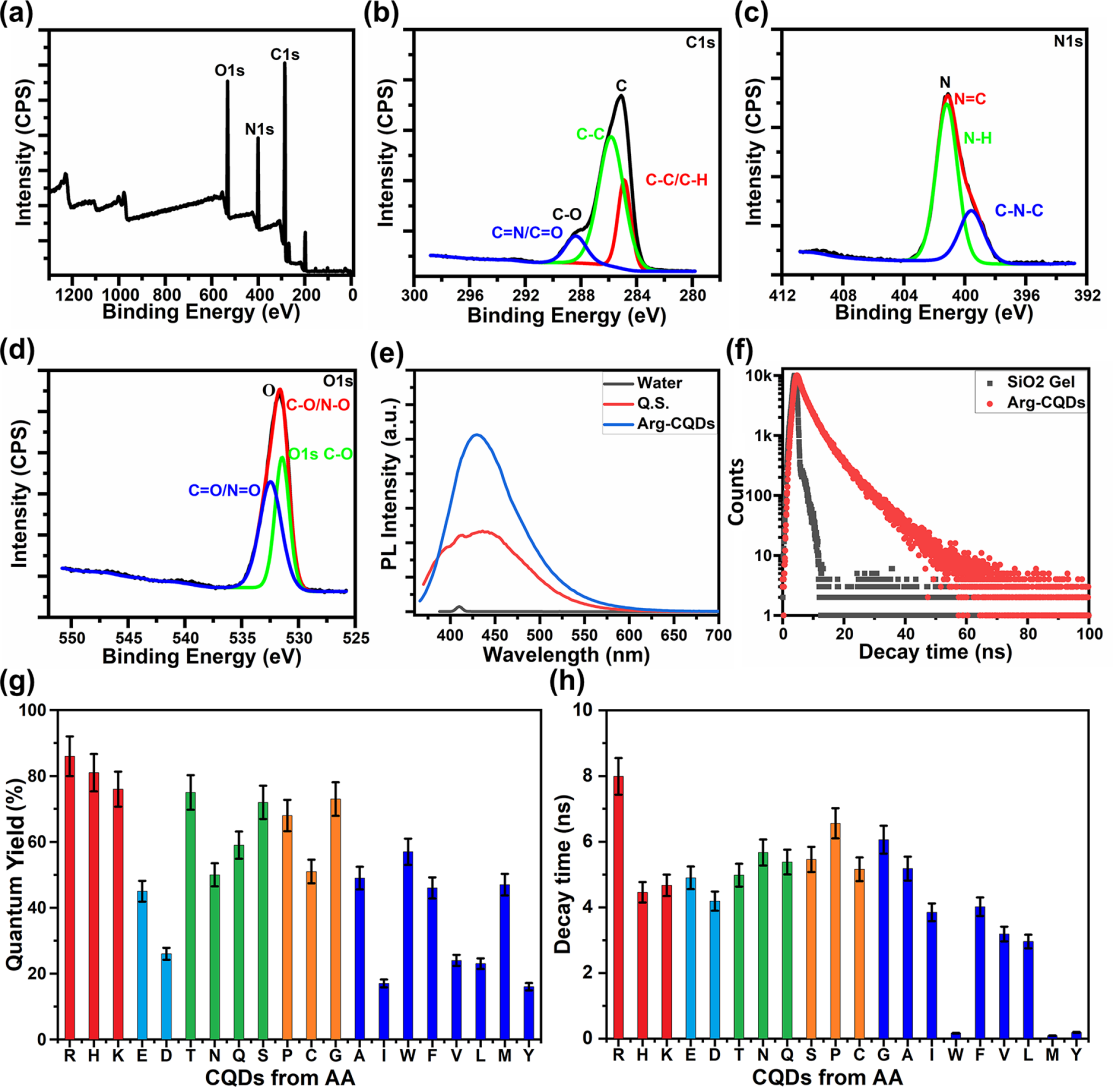
XPS analysis of AA-CQDs: (a) complete XPS spectrum and (b) C 1s, (c) N 1s, and (d) O 1s high-resolution spectra. (e) PL emission spectra of Arg-CQDs at λ_ext_ = 360 nm and quinine sulfate (QS, 0.1 mM). (f) PL decay time of Arg-CQDs on a logarithmic scale. (g) QYs of all 20 AA-CQDs, calculated using eq 1, based on the data shown in Figure S26. (h) PL decay times of all 20 AA-CQDs, based on the histograms shown in Figure S27: red, positively charged; cyan, negatively charged; green, uncharged side chain; orange, special cases; blue, hydrophobic side chain.

CQDs have become attractive materials in recent years, but their application has so far been limited by their low QY (often less than 20%).^49,50^ Several studies show that the QY of CQDs may be increased by N-doping.^49,51^ We therefore expected our CQDs to be effectively N-doped as well, since they are synthesized from AAs which contain nitrogen in the form of amines. XPS analysis confirmed that the CQDs were indeed doped with N. Figure 3a–e shows that carbon, nitrogen, and oxygen were present in the AA-CQD nanomaterials, with the presence of C 1s (65.8%), N 1s (17.5%), and O 1s (15.7%). As shown in Figure 3a, binding energy peaks were observed at 284, 399, and 531 eV, corresponding to the typical XPS survey peaks at C 1s, N 1s, and O 1s, respectively.^52,53^ A high-resolution C 1s spectrum is shown in Figure 3b, with peaks at 284.6, 285.7, 286.1, and 288.8 eV representing C=C, C–C, C–O/C–N, and C–O/C=N bonds, respectively.^22^ Based on the high-resolution N 1s spectrum, the binding energy peak at 399.9 eV indicated that pyridinic N was the dominant state (Figure 3c), indicating that some N was doped in defect areas or near the edges of graphitized carbon structures of the AA-CQDs. When nitrogen valence is analyzed, it has been observed that the percentages of N differ from one AA to other AA-based CQDs with an increase in dehydration and carbonization.^53^ The nitrogen in AA-CQDs is primarily present as pyridine nitrogen. This oxidative polymerization involves the amino nitrogen becoming the nitrogen on the six-membered ring-like pyridine nitrogen.^54^ Additionally, this indicates that the degree of dehydration and carbonization during the formation of AA-CQDs by hydrothermal reaction may contribute to their high fluorescence and high RI.^22,55^ This may be due to the reaction temperature and relatively high pressure, which aid in dehydration and carbonization and the conversion of pyridine nitrogen into pyrrole nitrogen. According to Figure 3d, the two peaks at 531.6 and 533.1 eV in the O 1s high-resolution spectrum represented C–O and C=O, respectively.^42^ There are slight differences in the percentages of the three elements in each of the 20 AA-based CQDs, but there is a higher amount of N in His-CQDs and Arg-CQDs compared to other AA-CQDs. High amounts of oxygen-containing functional groups (such as carboxyl groups) would cause large fluctuations in the optical properties of AA-CQDs. In addition, closer examination of the XPS analysis can reveal a significant degree of polarization due to the carboxyl groups, whose rotation relative to the basal plane affected the degree of polarization.^53^ Therefore, the oxygen content may be a result of the oxygen functional group present in AA-based CQDs and can also be responsible for affecting the RI of AA-CQDs.

### 2.4 Fluorescence QY and PL Decay Lifetime Characterizations

It was previously observed that AA-CQDs (i.e., N-doped CQDs) generally have higher QYs than pristine CQDs without N doping.^43^ As a result of our XPS analysis, we found that AA-CQDs contained more than 15% N, which is also indicative of a high QY. All AA-CQDs and quinine sulfate (QS) PL emission spectra were recorded at 360 nm in order to determine the integrated fluorescence intensity, which is defined as the area under the PL curve over the emission wavelength range of 370–700 nm. As the next step, the integrated fluorescence intensity was plotted against the absorbance value. In order to calculate QY values, the following equation was used
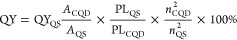
1where QY is the fluorescence quantum yield, *A* is the absorbance, PL is the fluorescence emission peak, and *n* is the RI, where the RI of the QS is 1.33. To reduce the effects of reabsorption, we kept the absorbance below 0.1 OD (optical density) in a 10 mm quartz cuvette. The fluorescence QYs of the AA-CQDs were estimated by comparing the integrated PL intensity (excited at 360 nm) and absorbance values (at 360 nm) to that of QS in a 0.1 mM H_2_SO_4_ solution (QY = 54%),^46^ as illustrated in Figures 3e and Figure S27. All AA-CQDs dispersed in water were transparent to white light for several weeks and remained stable. The fluorescence behavior of the AA-CQDs was influenced by their surface chemistry, such as N-doping and defect sites located on the surface, which trap excited-state energy, resulting in the highest QY values being 86%, 81%, 76%, 75%, 72%, 68%, and 73% for Arg-CQDs, His-CQDs, Lys-CQDs, Thr-CQDs, Ser-CQDs, Pro-CQDs, and Gly-CQDs, respectively. A summary of the QYs and other optical properties is presented in Table 1, and a graphical representation is shown in Figure 3g. According to this data, the QYs were influenced by the AA side chains. It is possible that this is due to the different functionalities of the chemical and electronic environments of AA-CQDs. A higher QY was observed for CQDs containing AAs with a positive charge compared with hydrophilic AAs. The long-chain polymer network formed a supramolecular self-assembled structure^56^ conducive to hydrophobic interactions, which is a key component of tunable optical properties. In addition, we were able to correlate the effect of the side chain volume to the emission of the AA-CQDs, and we found that as the side chain volume increased, the QY also increased. Notably, we observed that a higher percentage of heteroatoms (O, N, and S) in the CQDs correlated to a higher photoluminescence QY, up to 86%. These results indicate that heteroatoms play an important role in emission and improve the QY of AA-CQDs.

The fluorescence decay lifetime indicates how long the CQDs are in the excited state before they return to the ground state by emitting photons. The PL lifetime can only be measured using time-resolved spectrophotometers, which are commonly used to measure femtosecond laser pulses. The duration of the PL decay lifetime is also an important indicator when evaluating whether the obtained CQDs can be potentially implemented in the fields of solar cells, bioimaging, and sensing, particularly for intracellular applications. Different synthetic approaches and post-treatments affect the CQD QY, and different fabrication methods result in different PL decay lifetimes. Therefore, in order to widely utilize CQDs for bioimaging and sensing experiments, PL decay lifetimes must also be examined in addition to the QYs. We measured the PL decay lifetime of the 20 AA-CQDs to identify the best materials for the various applications. The PL properties of all 20 coded AA-CQDs such as QY and PL decay lifetime varied according to the passivation degree of the AA on the surface of the CQD, which was determined by the different functional groups of each AA (Figure S27). Comparatively, the Arg-CQDs exhibited the longest PL decay lifetime of 7.99 ns, presumably due to exhibiting the highest degree of amino passivation on their surfaces (Figures 3f). Figure 3h and Figure S28 show graphs of all coded AA-CQD decay times. In the PL decay time spectra (Figure S28), we can observe that Arg-CQDs, His-CQDs, Lys-CQDs, Glu-CQDs, Asp-CQDs, Thr-CQDs, Asn-CQDs, Gln-CQDs, Ser-CQDs, Pro-CQDs, Cys-CQDs, Gly-CQDs, Ala-CQDs, Ile-CQDs, Phe-CQDs, and Val-CQDs exhibited decay times of 3.0 ns (Table 1 and Figure 3h). In previous works that used a hydrothermal process, researchers prepared N-doped CQDs from different hydroxide biomolecules than we used^21,49,51,57^ and obtained QYs of ∼40–47% and PL decay lifetimes of 9 ns. Thus, our simply synthesized Arg-CQDs have higher QYs (86%) and PL decay times (∼8 ns) than those of any other conventional CQDs. Lastly, on the basis of the above figures and tables (Figure 3h and Table 1), the correlation among PL decay times, RI, and crystallinity in the HRTEM images vary for each AA-CQDs.

### 2.5 RI Measurement

The RI values of the AA-CQD suspensions were determined using a refractometer (ATAGO, PAL-RI) for a single wavelength of 598 nm and interferometric phase microscopy (IPM) for 8 different illumination wavelengths^58^ across the visible spectrum. For the IPM technique, the solutions were inserted into a 100 μm tall microfluidics channel (Ibidi, μ-Slide VI 0.1), and fluorescence microscopy imaging was performed to verify that the fluorescent AA-CQD suspension (fully transparent with no precipitation) had filled the channel. Next, off-axis holograms of the channel filled with the suspension were acquired using a shearing IPM system^58^ and a supercontinuum laser source (NKT SuperK EXTREME) coupled to an acousto-optical filter (NKT SuperK SELECT), thereby enabling acquisition of holograms with 8 different illumination wavelengths (490, 500, 515, 530, 620, 641, 650, and 680 nm).^58^ Additionally, background holograms of the channel containing only water were acquired for each wavelength. Following this, optical path delay (OPD) maps of the channel filled with each sample suspension and illuminated with each wavelength were reconstructed from their respective holograms, while using the corresponding background holograms to negate the OPD contribution of the water in the suspension. The OPD of a sample at a given point (*x*,*y*) and for an illumination wavelength λ is defined by
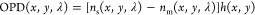
2where *n*_s_ is the RI of the sample, *n*_m_ is the RI of the surrounding medium (i.e., the medium of the background hologram), and *h* is the sample height. By rearranging the equation, we determined the RI of the suspension, *n*_s_, using the average OPD value of the channel in each hologram
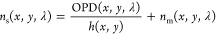
3where *h* is the known channel height, 100 μm, and *n*_m_ is the known RI of water for each wavelength, as previously published.^59^ Finally, the RI of the suspended AA was determined using the Lorentz–Lorenz mixture rule^60^
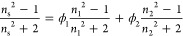
4where *n*_1_ and ϕ_1_ are the RI and the volume fraction of the suspended AA, respectively, and *n*_2_ and ϕ_2_ are the RI and the volume fraction of the suspension medium, respectively. The volume fractions were calculated using the known sample concentrations of the AA-CQDs and their density, and the values of *n*_2_ are identical to those of *n*_m_ from earlier.

**Figure 4:**
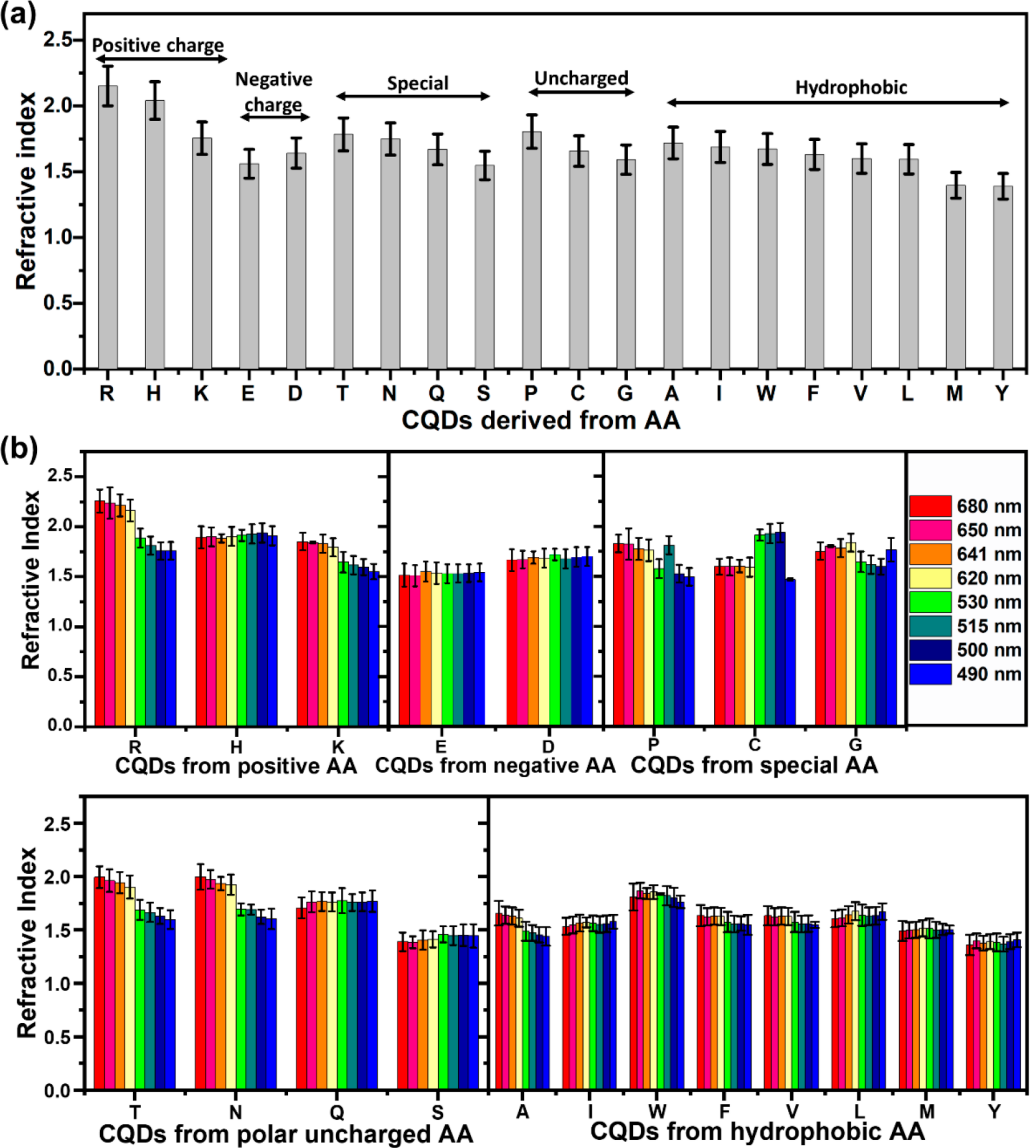
RIs of the AA-CQDs. (a) Refractometer RI measurements at a wavelength of 589 nm. (b) RIs of AA-CQDs at different wavelengths (680, 650, 641, 620, 530, 515, 500, and 490 nm) measured by the IPM technique.

The RI values of the AA-CQD suspensions were then determined. We found that our AA-CQDs possessed high RIs (>1.70), with RIs of 2.15 for Arg-CQDs, 2.04 for His-CQDs, 1.76 for Lys-CQDs, 1.79 for Thr-CQDs, 1.81 for Pro-CQDs, 1.75 for Asn-CQDs, and 1.72 for Ala-CQDs. The RI values of all AA-CQDs are shown in Figure 4a, grouped by charge, hydrophobicity, polarity, and aromaticity, as well as in Table 1. Additionally, using the IPM technique, we determined the RIs of all 20 AA-CQDs for illumination wavelengths of 490, 500, 515, 530, 620, 641, 650, and 680 nm (Figure 4b and Figures S30 and S31). Furthermore, we performed a control experiment to determine the RIs of aqueous solutions of pristine AAs and found that all 20 AAs had RIs <1.56. A graph of the RI values of the pristine coded AAs is shown in Figure S29. This high RI is likely caused by crystallization and the high compactness of the AA-CQDs, as we observed that these materials are highly crystalline with high fluorescence and QYs. In view of their high crystallinity, CQDs synthesized from AAs are expected to exhibit high RI and excitonic properties in bulk. Based on the measured extinction-dependent spectra (Figure 2 and Figure S26), we have observed a very fluorescence-rich structure, which is consistent with the predictions based on the high crystallinity from Figure 1 and the extended Mie theory for radially anisotropic AA-CQDs.^61^ As a result, AA-CQDs mimic the original crystal’s optical properties, which gives the possibilities for all-dielectric nanophotonics due to the very large dielectric permittivity of CQDs in the visible range, which surpasses that of traditional high-refractive-index materials. In light of the fact that the CQD nanomaterials possess high RI, they may enable control multipole moments and be able to effectively concentrate electromagnetic energy.^40,62^ Furthermore, the formation of high-quality colloidal AA-CQD crystals would result in optical diffraction at wavelengths based on the size of the sphere (or cavity) and the surface functionality, which also affects the RI of the materials. According to refractometer measurements and phase change measurements, Arg-CQDs, His-CQDs, Lys-CQDs, Pro-CQDs, and Thr-CQDs exhibit very high RI. At the same time, not all AA-CQDs nanomaterials of the same size display similar properties. As well as this, the high RI of CQDs leads to the excitation of Mie resonances that can be used to match windows of relative biological transparency (480–900 nm), which can also be applied to attractive theranostics applications.^63^ Furthermore, the HRTEM analysis of the synthesized AA-CQDs indicates a significantly high degree of crystallization and compactness, which may be one of the reasons for the high RI, though it is most likely that the high RI is due to the electromagnetic properties of these materials at the atomic scale.

Based on all of the above results, the fluorescence QY, RI, and crystallinity vary per AA-CQD. Some of the AA-CQDs (Arg-CQDs, His-CQDs, Lys-CQDs, Thr-CQDs, Ser-CQDs, Pro-CQDs, and Gly-CQDs) show high fluorescence, high QY, and high RI. The remaining 13 AA-CQDs do not present clear correlations of high fluorescence, high RI, and long decay time. All AA-CQD materials also displayed excitation-dependent fluorescence across a wide range of the electromagnetic spectrum, photostability, and water-solubility.

### 2.6 *In Vitro* Cytotoxicity and Cellular Imaging Using the AA-CQDs

**Figure 5:**
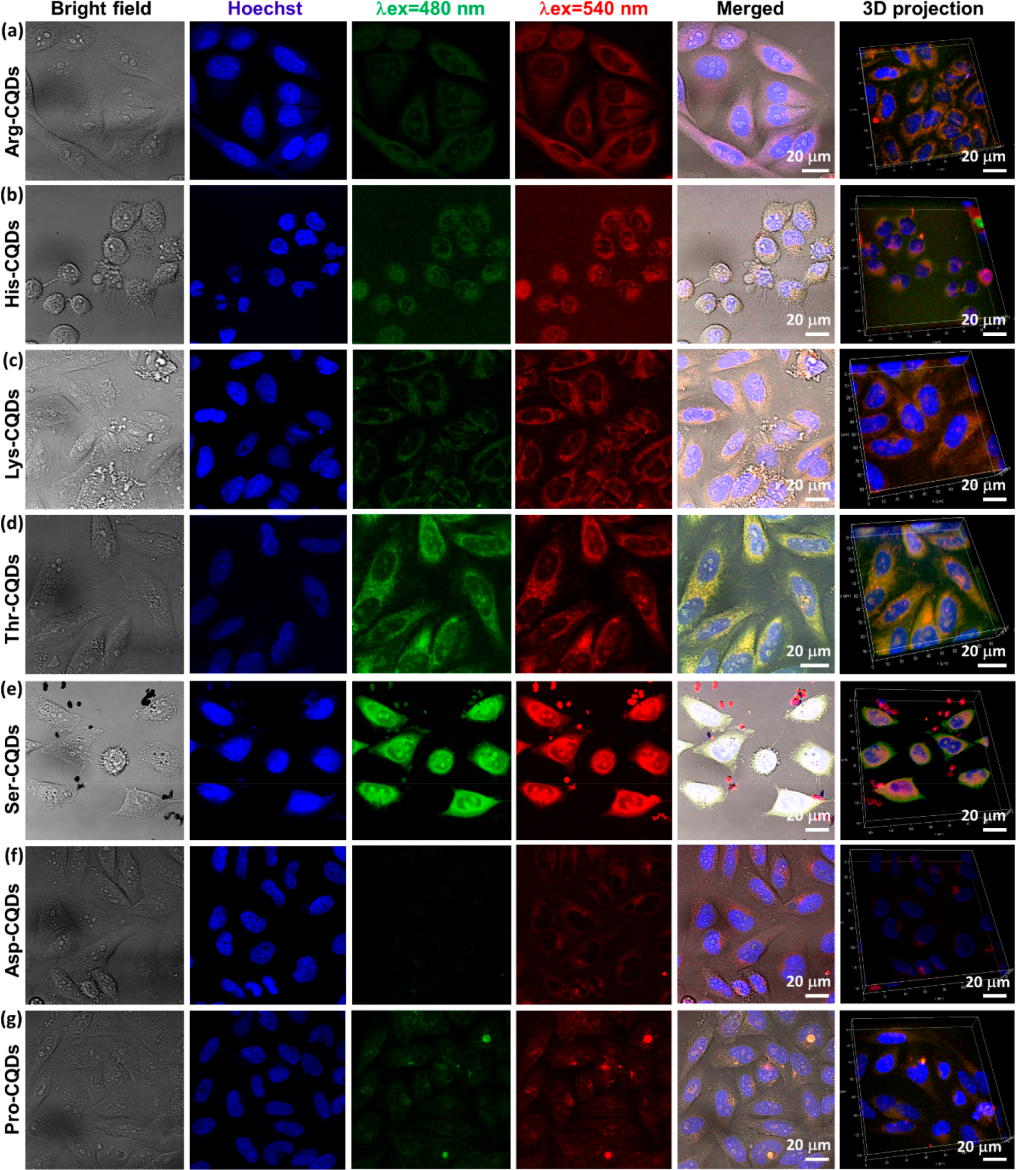
Live cell imaging of AA-CQDs in Hela cells (a) Arg-CQDs, (b) His-CQD, (c) Lys-CQDs, (d) Thr-CQDs, (e) Ser-CQDs, (f) Asp-CQDs, and (g) Pro-CQDs images using four fluorescence channels: bright field images, Hoechst (dye staining live cell nucleus), excitation at 480 nm, and excitation at 540 nm. A z-stack taken by a confocal microscope provides the 3D-projection showing the Hela cell with corresponding CQD nanomaterials. Scale bar: 20 μm.

AA-functionalized surfaces are the most straightforward method of mimicking biological surfaces for biocompatible nanomaterials. Their low toxicity and high biocompatibility imply promising potential for use in biomedical applications such as cellular imaging, drug delivery, and nucleolus-targeted tracking. Thus, we set out to quantify the biocompatibility of all 20 AA-CQDs. For this purpose, an MTT assay was conducted on human breast cancer cells (HeLa) to assess the cytotoxicity (Figure S32) of all 20 coded AA-CQDs at different concentrations (0.50, 100, 200, and 500 μg/mL). The assays showed that over 95% of the cells survived after a 24 h treatment with AA-CQDs at a concentration of 100 μg/mL, indicating excellent biocompatibility. Following the cytotoxicity assays, we examined the live cell bioimaging capabilities of the most fluorescent AA-CQDs (QY > 45%): His-CQDs, Arg-CQDs, Lys-CQDs, Thr-CQDs, Ser-CQDs, Asp-CQDs, and Pro-CQDs. Using a confocal microscope in conjunction with Hoechst dye for nucleus localization, we observed AA-CQD fluorescence residing within the cytoplasm (Figure 5a–g), near the nuclear membrane (Figure 5a–d), and partially within the nucleus (Figure 5d,e). These results indicate that Thr-CQDs, Ser-CQDs, Arg-CQDs, Lys-CQDs, and His-CQDs can be excellent agents for targeted nuclear membrane tracking and partial nuclear penetration. Comparing the results of the AA-CQDs to the control (without any treatment with AA-CQDs, Figure S33), the AA-CQDs bind efficiently to the nuclear membrane and also enter the nucleus of the cell. These AA-CQDs display fluorescence at three fluorescence channels, with λ_ex_ = 405, 480, 540 nm and λ_em_= 420–470, 500–580, and 560–630 nm, respectively. Specifically, three fluorescence excitation channels, 405, 480, and 540 nm, displayed optimal performance. We found that depending on the surface composition, the AA-CQDs could target the nucleolus and cross the nuclear membrane. According to the above cellular analysis, we have observed that positively charged AA-CQDs and polar uncharged AA-CQDs are more suitable for cellular imaging due to their high fluorescence and QYs, as well as their high RI.^64^ The AA-CQDs are also suitable for biomedical applications as they are noncytotoxic, and we have found that CQDs are excellent for live cell imaging with very high contrast.^5,64,65^ Taken together, AA-CQD materials are among the highest RI carbon quantum materials applicable for optoelectronic devices, bioimaging, biosensing, and the study of cellular organelles *in vivo*. This exceptional RI allows not only high sensitivity detection of cellular elements but also the differentiation between elements of different densities.

## 3 Conclusions

We have developed multifunctional CQDs derived from AAs showing tunable core and surface properties and investigated their use for the design of high-RI materials for cellular imaging and biomedical applications. The AA-derived N-containing CQDs are especially interesting due to their high QY and potential biomedical applications. We obtained the highest RI (∼2.1) ever reported for any CQD with our Arg-CQDs, along with high fluorescence and high QY (∼86%) and a long decay time (∼8.0 ns) compared to all other AA-derived CQDs. Moreover, we have demonstrated that all 20 AA-based N-containing CQD materials show excitation-dependent emission profiles (from UV to visible light), are photostable, water-soluble, and noncytotoxic, and are excellent for imaging live cells. In order to fabricate biocompatible nanomaterials, AA-functionalized surfaces are the most straightforward method of mimicking biological surfaces. Their high biocompatibility is manifested in their low toxicity toward HeLa cells as demonstrated herein. We also found that the AA-CQDs can target the nucleolus and cross the nuclear membrane depending on their surface composition. In conclusion, our results indicate that N-containing CQD materials derived from AAs are well-suited for designing high-RI materials for optoelectronic devices, bioimaging, biosensing, and manipulation of cellular organelles *in vivo*.

## 4 Experimental Methods

### 4.1 Required Materials

Quinine sulfate (98.0%), All 20 AAs (arginine, histidine, lysine, aspartic acid, glutamic acid, serine, threonine, asparagine, glutamine, cysteine, glycine, proline, alanine, valine, isoleucine, leucine, methionine, phenylalanine, tyrosine, and tryptophan) were purchased from Sigma-Aldrich Co., Ltd. In this study, we used 20 AAs of high purity with a purity level greater than 99.0%. No further purification was performed on the materials.

### 4.2 Synthesis of Water-Soluble CQDs

We prepared CQD nanomaterials using our modified hydrothermal method described previously.^66^ Briefly, 100 mg of AA was dissolved in 20 mL of deionized water (DIW) separately, to avoid cross-contamination. This was then transferred to a 50 mL Teflon-lined autoclave and heated at 200 °C for 6–10 h in a hot air oven (as indicated in Figure S1). In most cases, a dark yellow-brown liquid was obtained after the autoclave was cooled to 25 °C. A small amount of carbide slag was removed from the product solution by Millipore filtration (0.22 m). The solution was then dialyzed with DIW using Amicon Ultra-15 Centrifugal Filter Units (MWCD = 2000 Da) to remove the unreacted AAs. The resulting aqueous solution of N-containing AA-CQDs was then evaluated for fluorescence, RI, decay time, physical morphology, chemical behavior, cell viability, and live cell imaging for the different AA-CQDs.

### 4.3 Analytical Methods

In order to perform the fluorescence spectroscopy measurement, 2 mL of a sample solution was pipetted into a four-sided transparent quartz cuvette with a path length of 10 mm, at ambient temperature, and the spectrum was collected using a FluoroMax-4 Spectrofluorometer (Horiba Jobin Yvon, Kyoto, Japan). Excitation and emission wavelengths were set at 320–540 and 350–900 nm, respectively, each with a 2 nm slit width. The AA-CQDs were analyzed with a spectrophotometer (Varian Cary 100) under ultraviolet (UV) light. Transmission electron microscopy (HR-TEM, JEOL 2100) was used to analyze the morphology and crystalline properties of the CQDs. In order to determine the chemical composition of the AA-CQDs, energy dispersive X-ray spectroscopy (EDS) was used as well as selected area electron diffraction (SAED) for crystallography information. The two analyses (EDS and SAED) were conducted using the same TEM analytical devices. After the CQD materials were dried using lyophilization, an X-ray photoelectron spectroscopy (XPS) analysis was conducted. We used an ESCALAB QXi X-ray Photoelectron Spectrometer Microprobe to record XPS of AA-CQDs using a monochromatic X-ray source (microfocused dual-anode Al K-Alpha and Ag L-Alpha source) with Al Kα excitation (1486.6 eV) and C 1s as the reference energy (EC 1s = 284.0 eV).

## Supporting Information Available

Methods and experimental details, figures including normal camera, TEM images, fluorescence of spectra, quantum yield spectrum, and refractive index of AA-CQDs, data on cell viability, and information regarding the live cell imaging of control cell (PDF)
