# Supplementary material for: High Quantum Yield Amino Acid Carbon Quantum Dots with Unparalleled Refractive Index
Source: ACS Nano. 2024 Jan 8;18(3):2421–33. doi: 10.1021/acsnano.3c10792 (PMC10811667; doi:10.1021/acsnano.3c10792)
Supplement: Supplementary file 1 — nn3c10792_si_001.pdf [file nn3c10792_si_001.pdf]

Supporting information

High Quantum Yield Amino Acid Carbon Quantum Dots with Unparalleled Refractive Index

Vijay Bhooshan Kumar[1], Simcha K. Mirsky[2], Natan T. Shaked[2], Ehud Gazit[1,3*]

[1]The Shmunis School of Biomedicine and Cancer Research, George S. Wise Faculty of Life Sciences, Tel Aviv University, 6997801, Tel Aviv, Israel
[2]Department of Biomedical Engineering, Iby and Aladar Fleischman Faculty of Engineering, Tel Aviv University, Tel Aviv 6997801, Israel
[3]Department of Materials Science and Engineering, Iby and Aladar Fleischman Faculty of Engineering, Tel Aviv University, Tel Aviv 6997801, Israel
*Corresponding Author: Ehud Gazit; ehudga@tauex.tau.ac.il

1. Experimental Methods

1.1 Quantum yields (QY): QYs of all AA-CQDs were determined by comparing their photoluminescence (PL) intensities (excited at 360 nm) and absorbance values (at 360 nm) to that of quinine sulfate (QS) in a 0.1 mM H_2SO_4 solution (QY = 54%). All AA-CQDs and QS PL emission spectra were recorded at 360 nm in order to determine the integrated fluorescence intensity, which is defined as the area under the PL curve over the wavelength range of 370 to 700 nm. As a next step, the integrated fluorescence intensity was plotted against the absorbance value. In order to calculate QY values, the following equation was used:

$$QY = QY_{QS}\frac{A_{CQD}}{A_{QS}} \times \frac{PL_{QS}}{PL_{CQD}} \times \frac{n_{CQD}^2}{n_{QS}^2} \times 100\% \quad (1)$$

where QY is the fluorescence quantum yield, A is the absorbance, PL is the fluorescence emission peak, and n is the RI, where the RI of the quinine sulphate is 1.33. To reduce the effects of re-absorption, we kept the absorbance below 0.1 OD (optical density) in a 10 mm quartz cuvette.

1.2 Fluorescence decay time: AA-CQD suspensions were placed in quartz cuvettes and the fluorescence/PL decay time was measured using a FluoroMax-4 Spectrofluorometer (Horiba Jobin Yvon, Kyoto, Japan). This spectrometer is equipped with double monochromators, a 450 W Xe lamp, an AGILE source, time-correlated single photon counting capability, a standard cuvette holder, and a standard PMT-900 detector. Fluoracle's standard option for acquiring decay times based on excitation provides automatic acquisition of decay times based on

excitation. A standard streak camera (Hamamatsu) was used in conjunction with a femtosecond laser excitation source able to produce several wavelengths of excitation to measure the excitonic decay lifetime of all twenty AA-CQDs. In this study, we measured time-resolved PL at wavelengths of 400 and 440 nm.

1.3 Refractive index measurements: The RI of the AA-CQD suspensions were determined by refractometer (ATAGO, PAL-RI), for a single wavelength of 598 nm, and by interferometric phase microscopy (IPM) for 8 different wavelengths across the visible spectrum. For the IPM technique, the solutions were inserted into a 100 μm tall microfluidic channel (Ibidi, μ-Slide VI 0.1) and fluorescence microscopy imaging was performed to verify that the fluorescent AA-CQD suspension (fully transparent and with no precipitation) had filled the channel. Next, off-axis holograms of the channel filled with the suspension were acquired using our shearing IPM system[37] and a supercontinuum laser source (NKT SuperK EXTREME) coupled to an acousto-optical filter (NKT SuperK SELECT), thereby enabling acquisition of holograms with 8 different illumination wavelengths (490, 500, 515, 530, 620, 641, 650, and 680 nm).[37] Additionally, background holograms of the channel containing only water were also acquired for each wavelength. Following this, optical path delay (OPD) maps of the channel filled with each sample suspension and illuminated with each wavelength were reconstructed from their respective holograms while using the corresponding background holograms to negate the OPD contribution of the water in the suspension. The OPD of a sample at a given point *(x,y)* and for an illumination wavelength λ is defined by:

$$OPD(x,y,\lambda) = [n_s(x,y,\lambda) - n_m(x,y,\lambda)]h(x,y) \quad (2)$$

where n_s is RI of the sample, n_m is the RI of the surrounding medium (i.e. the medium of the background hologram), and h is the sample height. By rearranging the equation, we determined the RI of the suspension, n_s, using the average OPD value of the channel in each hologram, as follows:

$$n_s(x,y,\lambda) = \frac{OPD(x,y,\lambda)}{h(x,y)} + n_m(x,y,\lambda) \quad (3)$$

where h is the known channel height, 100 μm, and n_m is the known RI of water for each wavelength, taken from literature.[38] Finally, the RI of the suspended AA was determined using the Lorentz-Lorenz mixture rule[39]

$$\frac{n_s^2 - 1}{n_s^2 + 2} = \phi_1 \frac{n_1^2 - 1}{n_1^2 + 2} + \phi_2 \frac{n_2^2 - 1}{n_2^2 + 2} \quad (4)$$

where n_1 and Φ_1 are the RI and the volume fraction of the suspended AA, respectively, and n_2 and Φ_2 are the RI and the volume fraction of the suspension medium, respectively. The volume

fractions were calculated using the known sample concentrations of the AA-CQDs density, and the values of n_2 are identical to n_m from earlier.

1.4 Cell Viability Measurement: A methyl thiazolyl tetrazolium (MTT) assay was performed using human cervical carcinoma cells (HeLa cells) to assess the cytotoxicity of AA-CQD nanomaterials. The cells were seeded into 96-well microtiter plates and allowed to adhere overnight at 37 °C under 5% CO_2. Following 24 hours of HeLa cell growth, all cell culture media was removed, and the cells were treated with AA-CQD nanomaterials without FBS (fetal bovine serum) medium. After this, the cells were washed with 7.4 pH phosphate buffer solution (PBS) and incubated with MTT solution for 3.5 hours. Following that, the MTT reagent was discarded, and 100 µL of DMSO was added to each well in order to dissolve the formazan crystals. We measured the absorbance at 570 and 680 nm using a microplate reader. Control experiments were conducted without AA-CQD nanomaterials. The experiments were conducted in quadruplicate.

1.5 Live cell imaging: The live cell imaging of Hela cells was conducted using confocal microscopy after the cells were grown in glass bottom petri dishes. HeLa cells were cultured in high glucose media for two days, then 100 µg/mL of AA-CQDs were added into the cell culture media and incubation was continued for different lengths of time (2h, 6h and 24h). The imaging was performed using an SP8 inverted confocal microscope (Leica Microsystems, Wetzlar, Germany). The excitation and emission ranges were as follows: λ_{ex} = 480 nm, 543 nm, 630 nm, and λ_{em} = 500–580 nm, 560-630 nm, and 650-740 nm, respectively.

2. Results

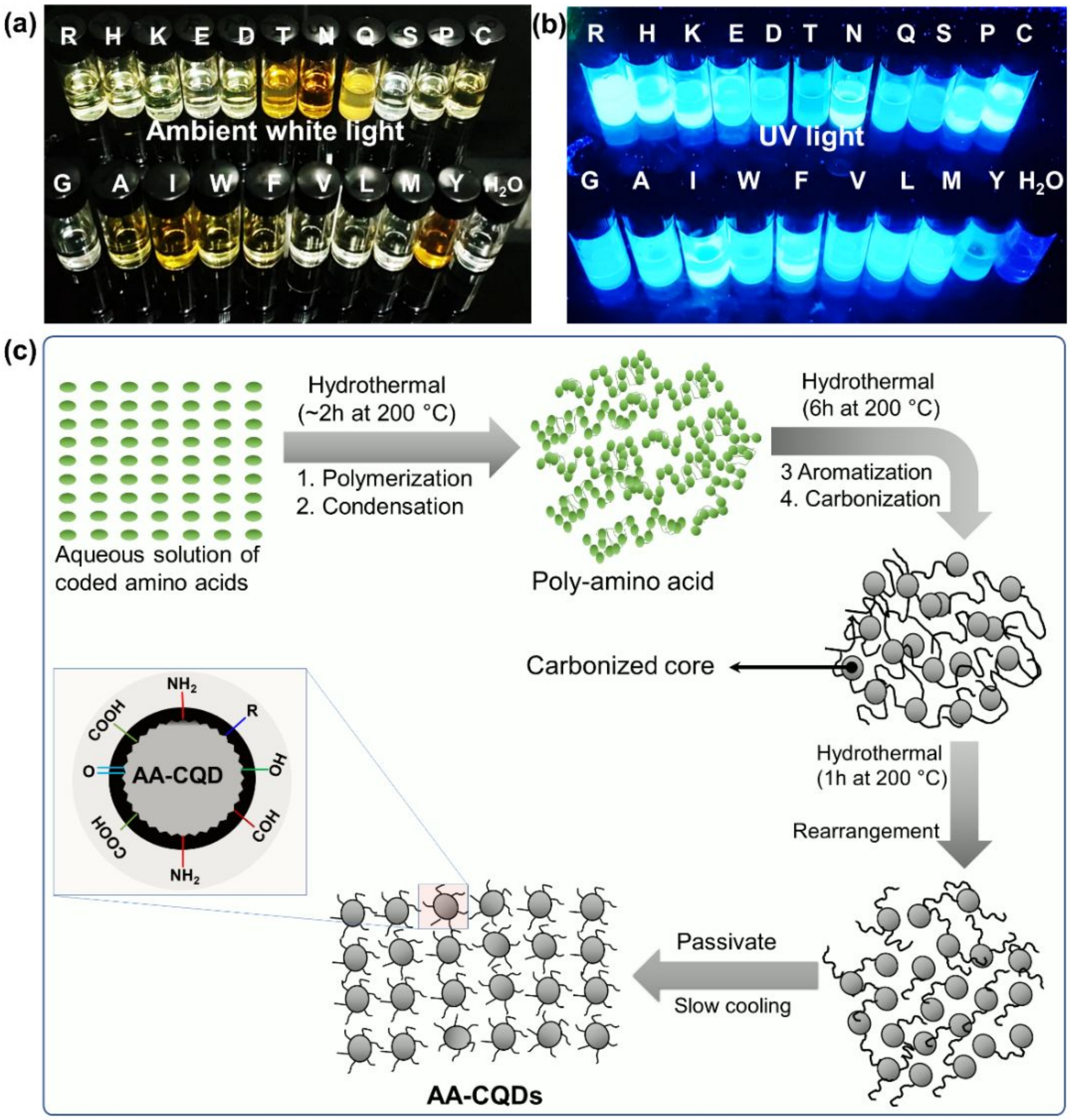

Figure S1. Visual analysis of pristine AAs and AA-CQDs under white and UV light. (a) AA-CQDs under ambient white light. (b) AA-CQDs from (a) under UV light. Associated AA and AA-CQDs label letters are in white. (c) Schematic representation of a possible mechanism for the formation of AA-CQDs from AAs through dehydration, polymerization, carbonization and passivation.

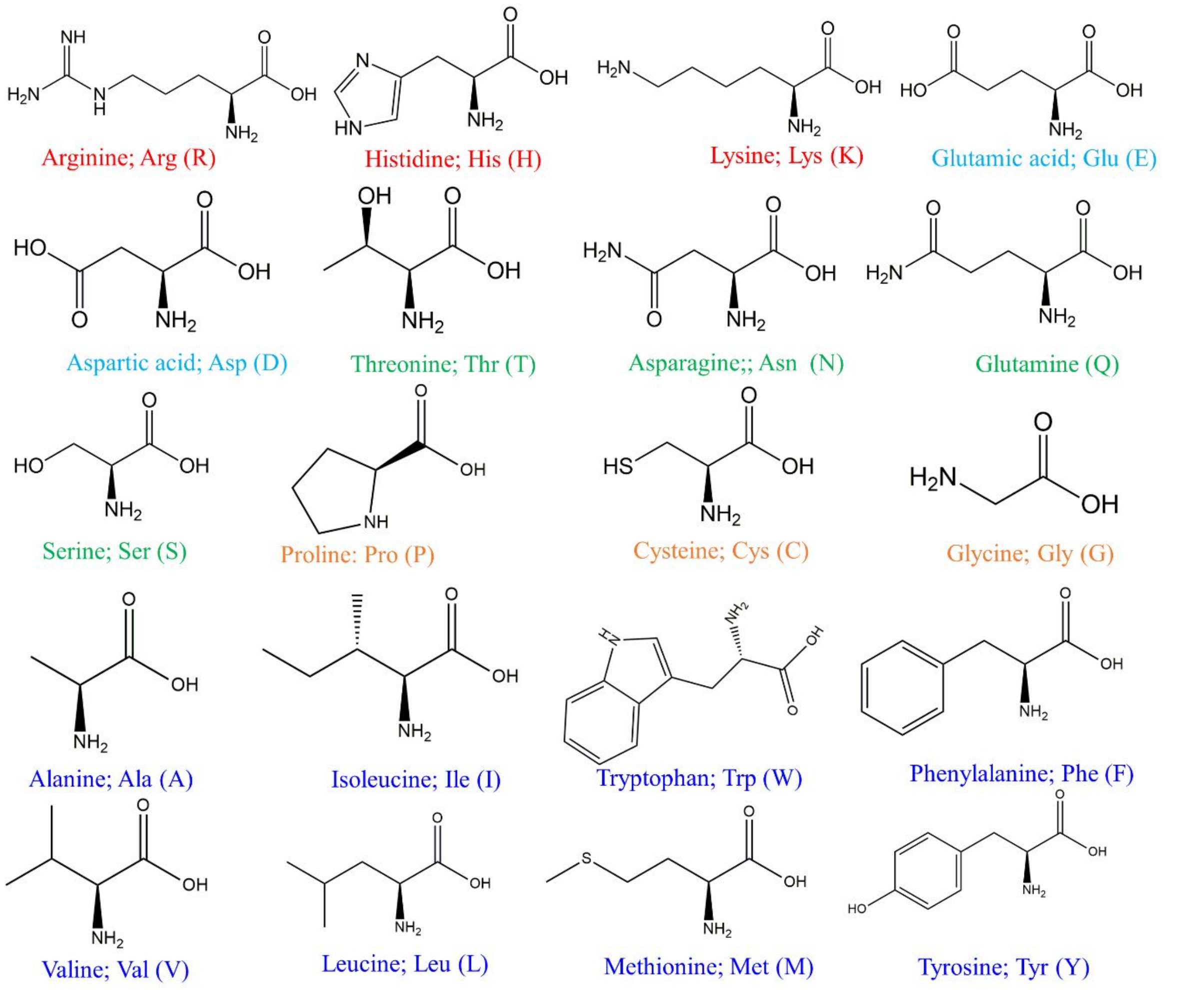

Figure S2: The scheamatic reperesentation of chemical formula of all the amino acids, which were used for the synthesis of CQDs from all the amino acids.

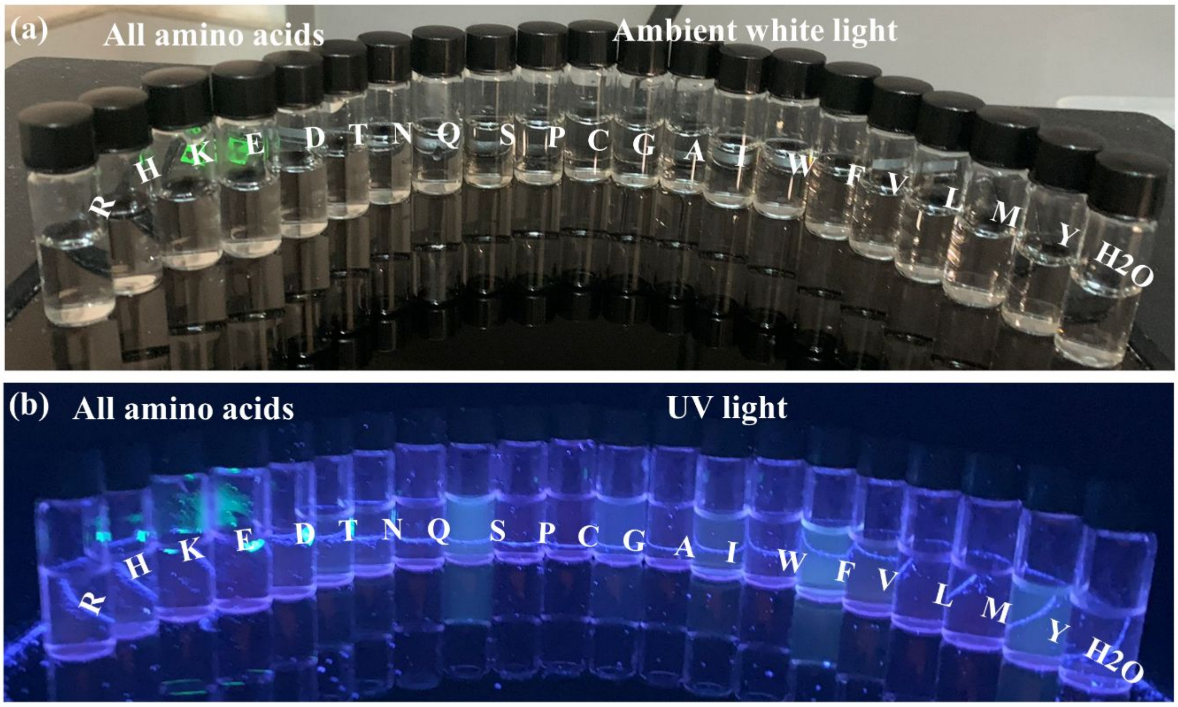

Figure S3. Visual analysis of pristine AAs and AA-CQDs under white and UV lights. (a) Aqueous solutions of pristine AAs under ambient white light. (b) Solutions from (a) under UV light. Associated AA label letters are in green for AAs and in red for AA-CQDs.

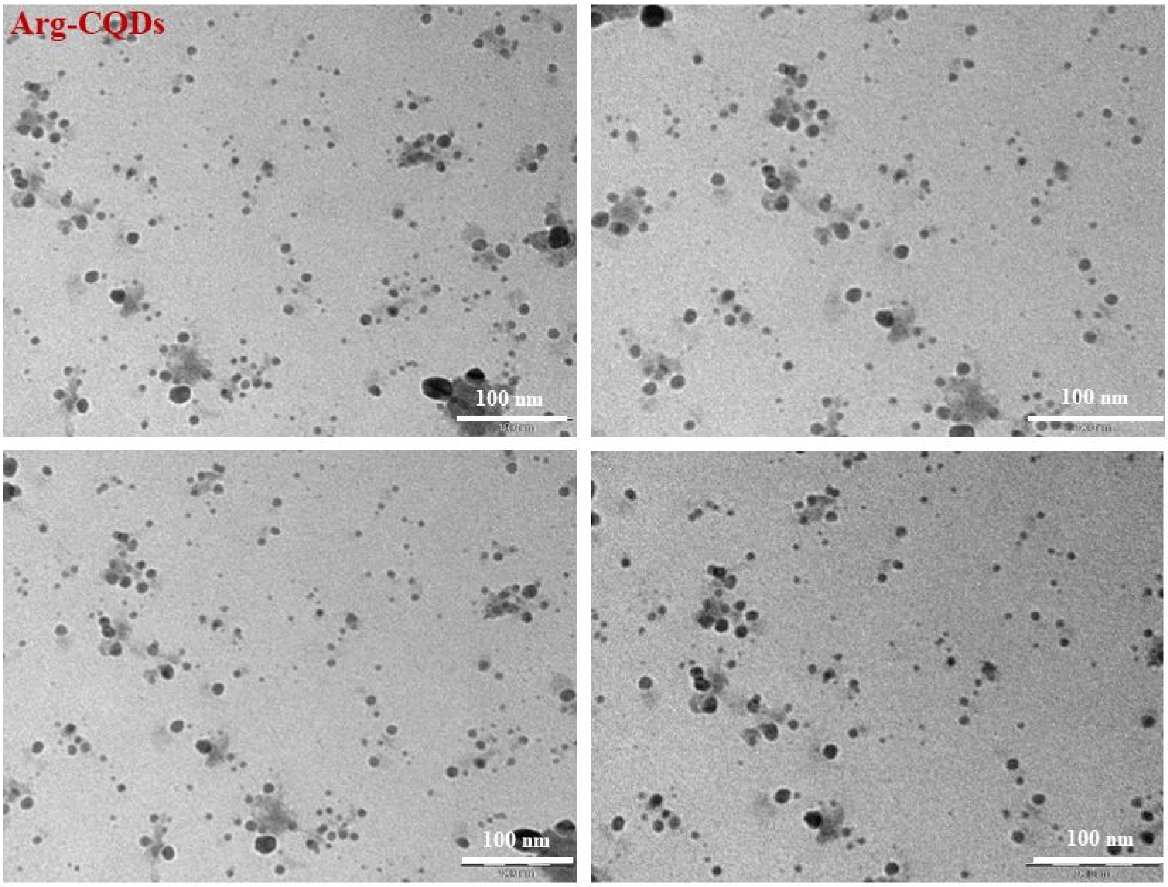

Figure S4: TEM micrograph of Arginine derived CQDs (Arg-CQDs) from arginine molecules.

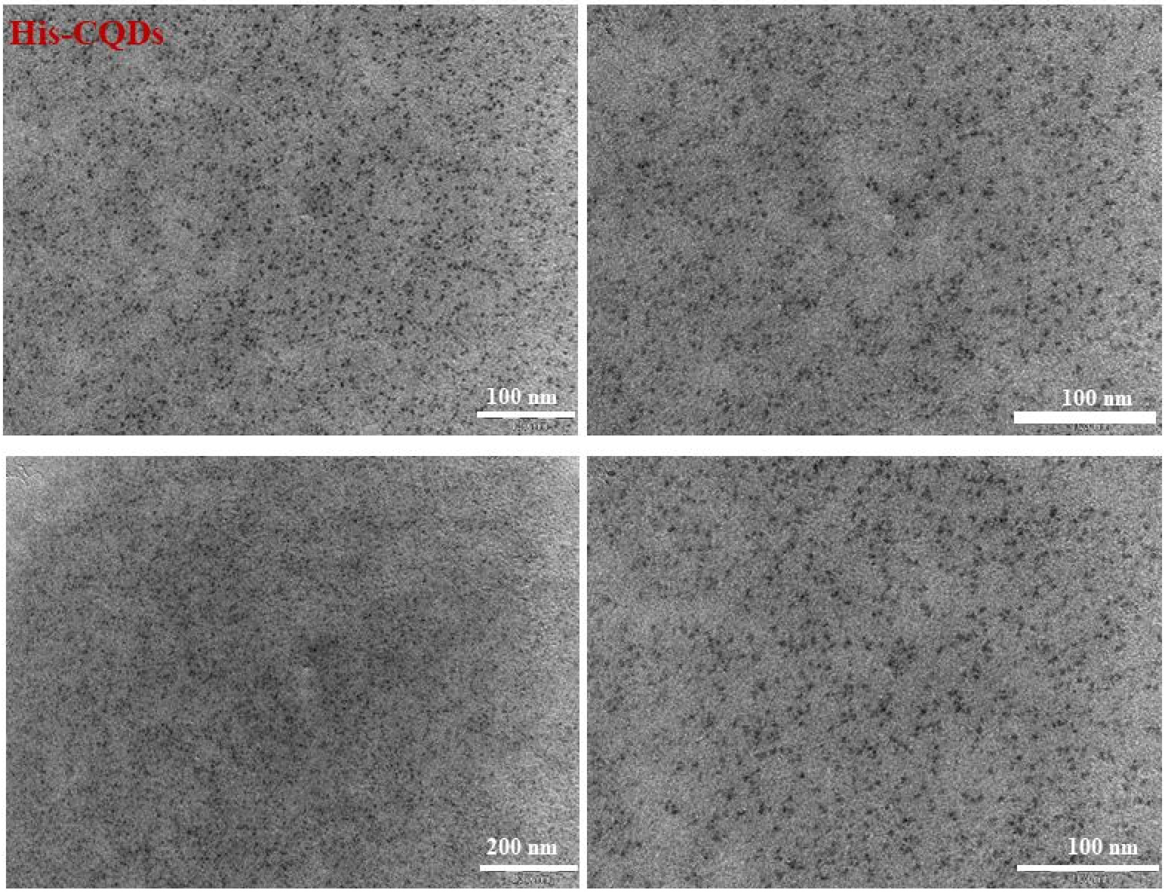

Figure S5: TEM micrograph of histidine derived CQDs (His-CQDs) from histidine molecules.

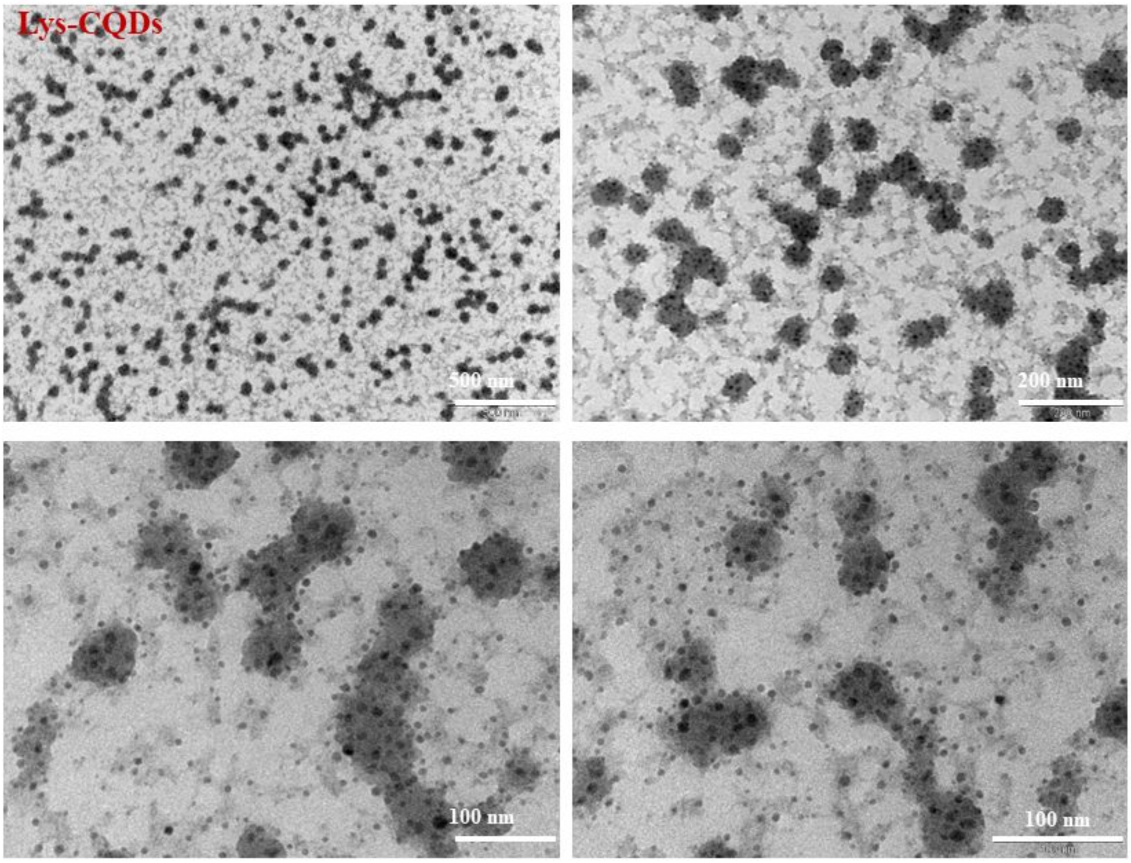

Figure S6: TEM micrograph of lysine derived CQDs (Lys-CQDs) from lysine molecules.

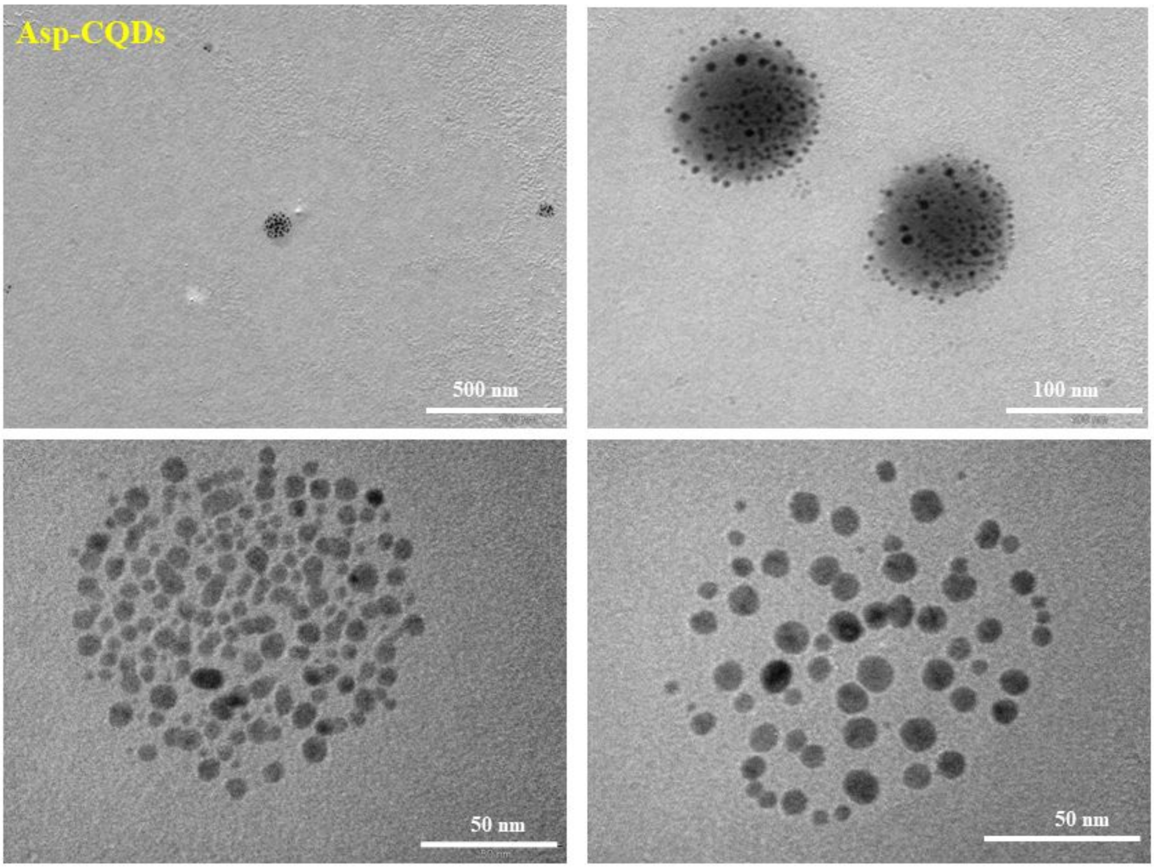

Figure S7: TEM micrograph of aspartic acid derived CQDs (Asp-CQDs) from aspartic acid molecules.

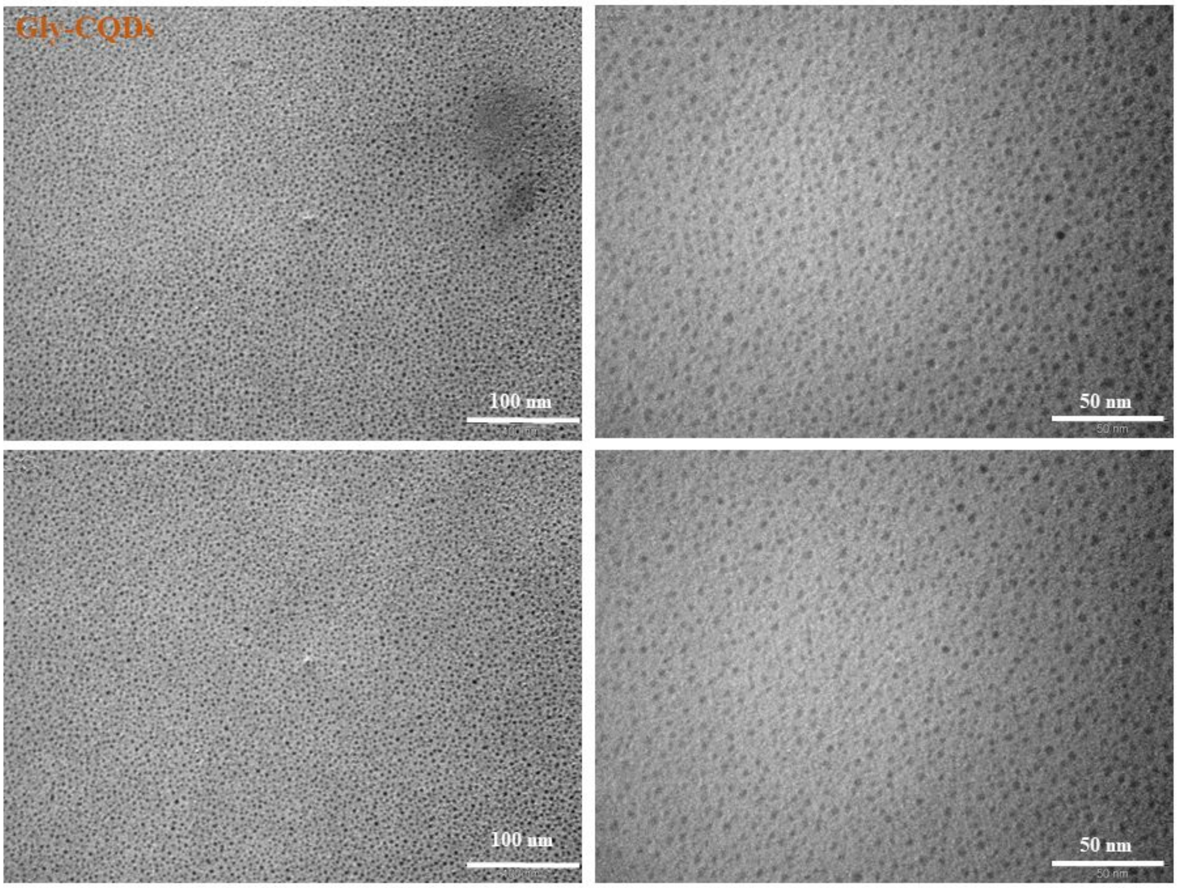

Figure S8: TEM micrograph of Glycene derived CQDs (Gly-CQDs) from glycene molecules.

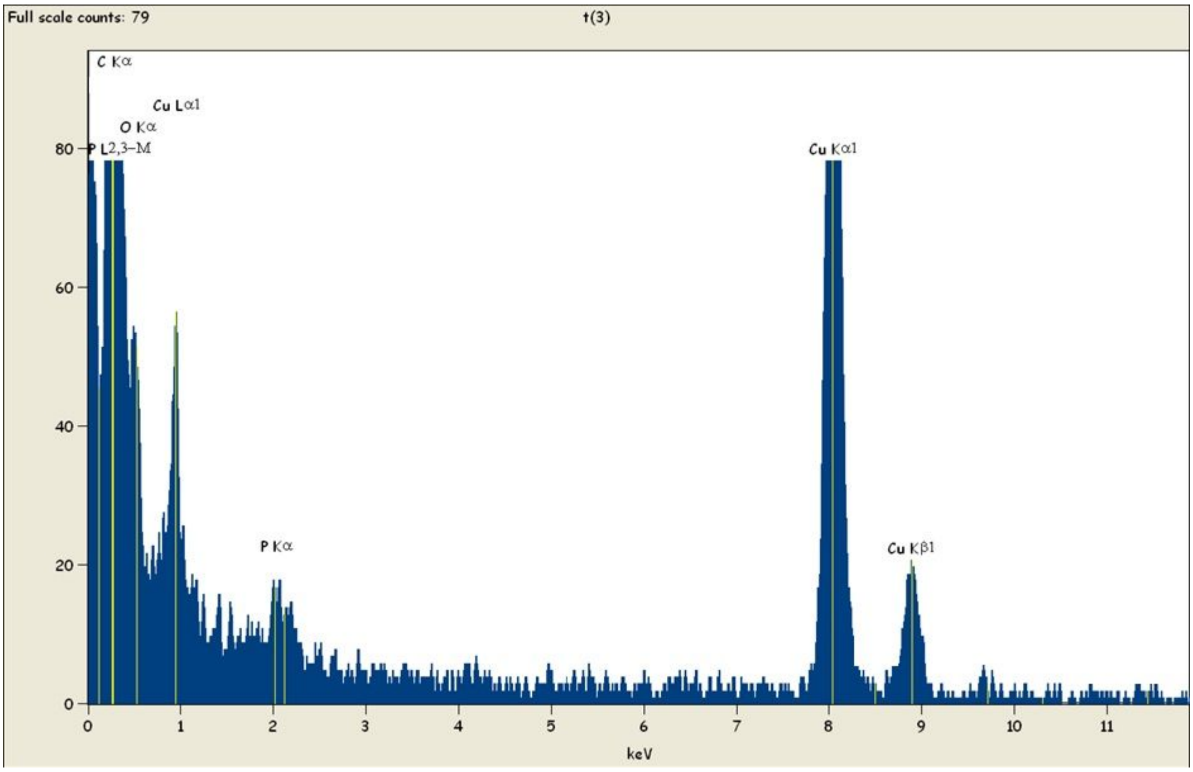

Figure S9: EDS spectrum of His-CQDs materials.

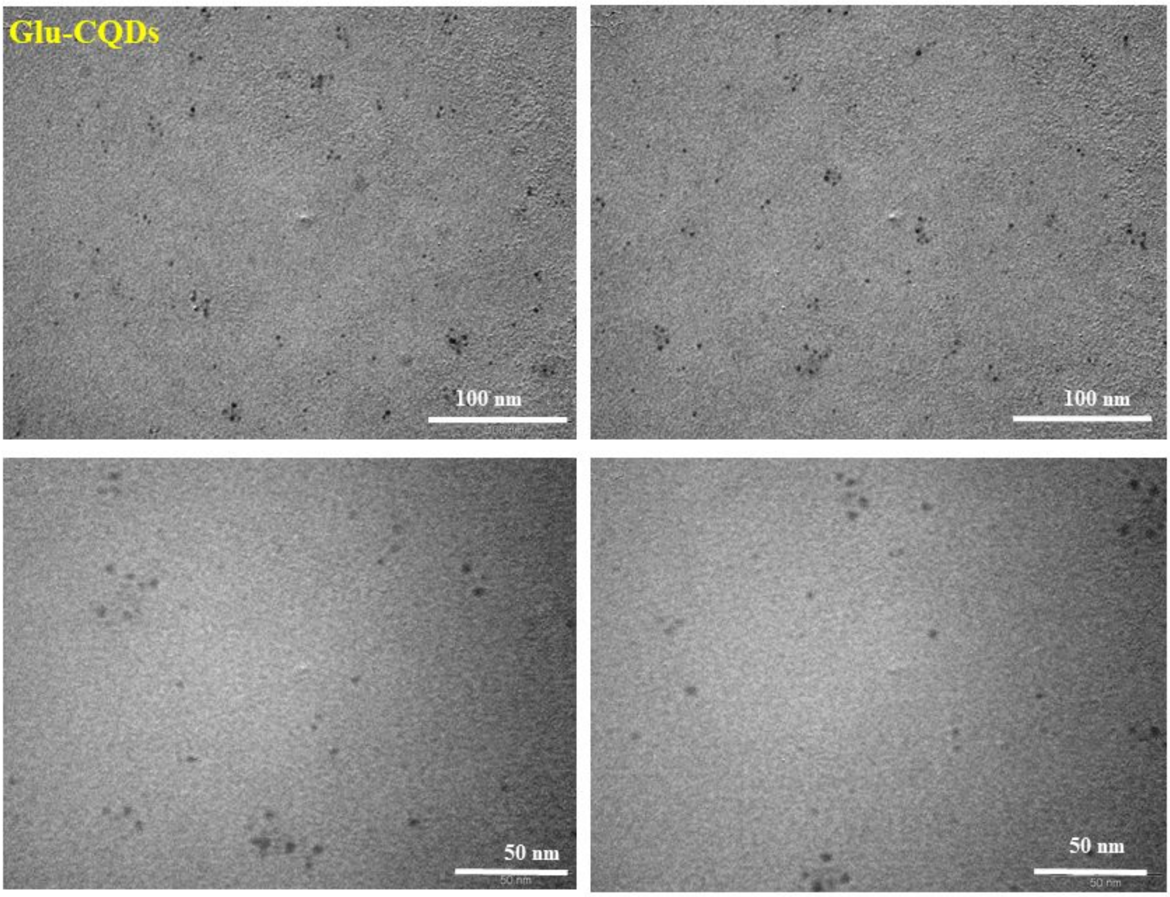

Figure S10: TEM micrograph of glutamic acid derived CQDs (Glu-CQDs) from glutamic acid molecules.

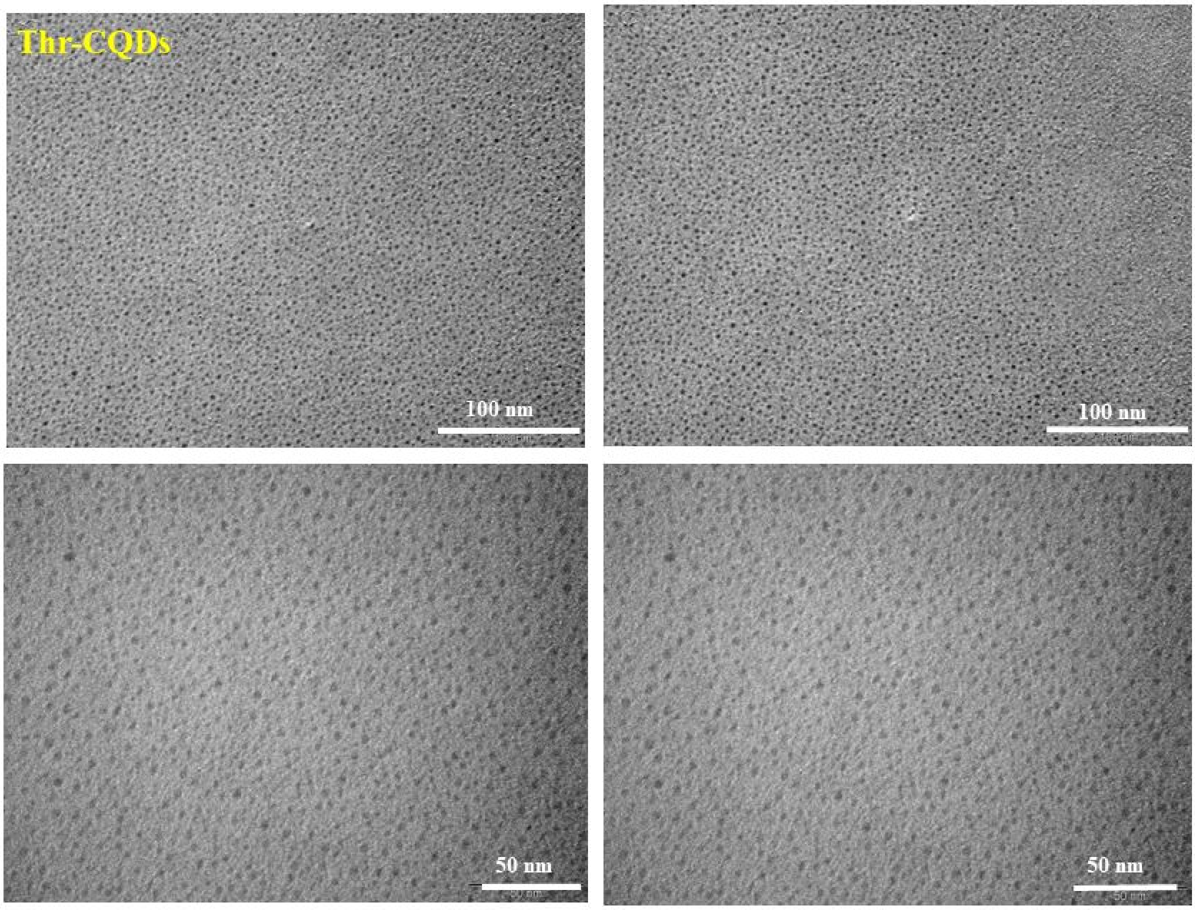

Figure S11: TEM micrograph of theronine derived CQDs (Thr-CQDs) from threonine molecules.

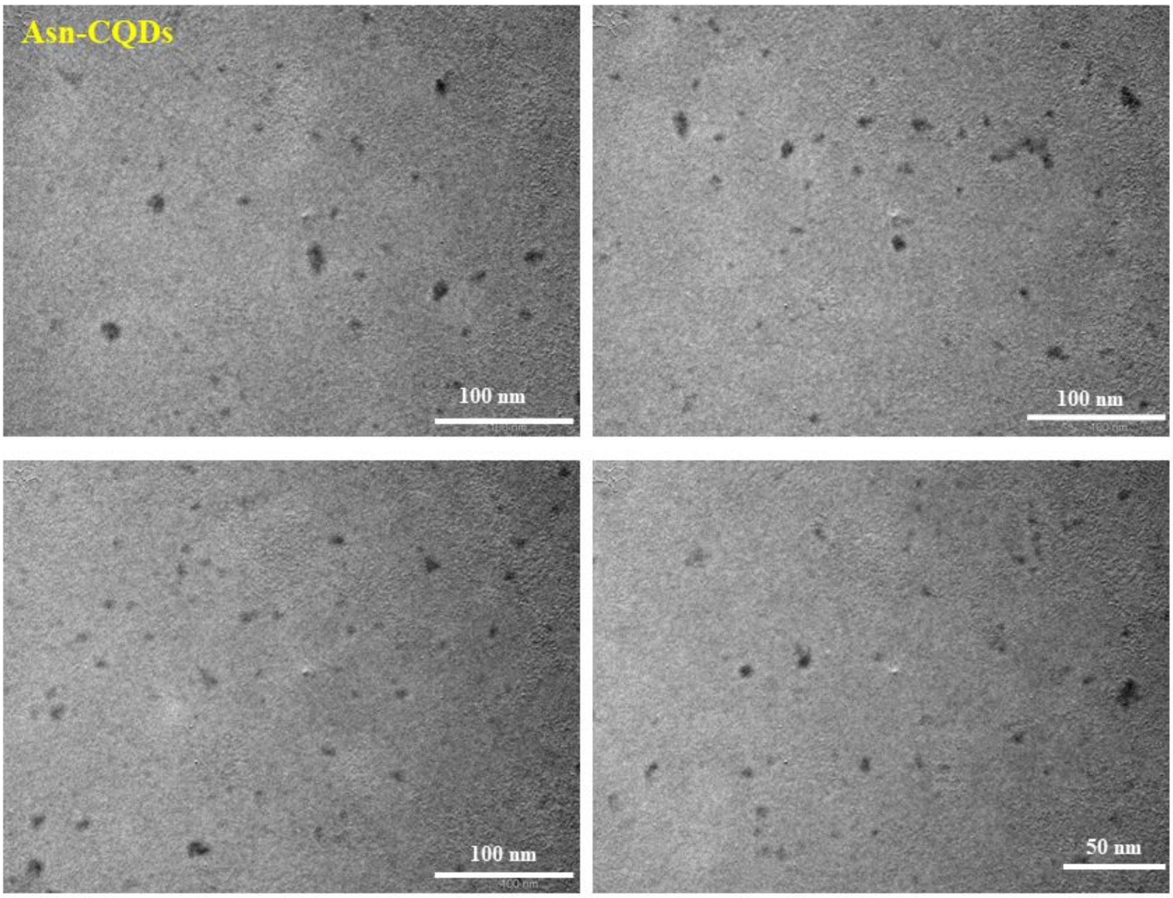

Figure S12: TEM micrograph of asparagine derived CQDs (Asn-CQDs) from asparagine molecules.

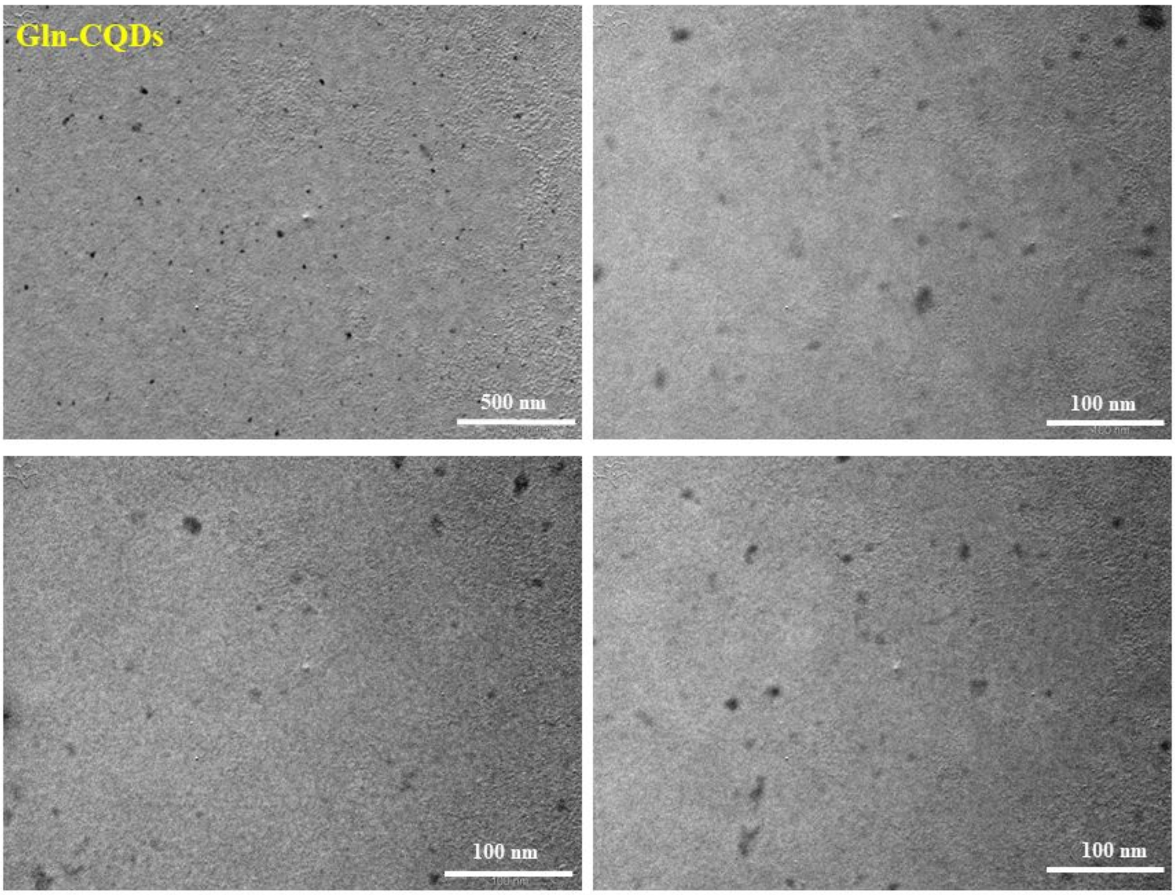

Figure S13: TEM micrograph of glutamine derived CQDs (Gln-CQDs) from glutamine molecules.

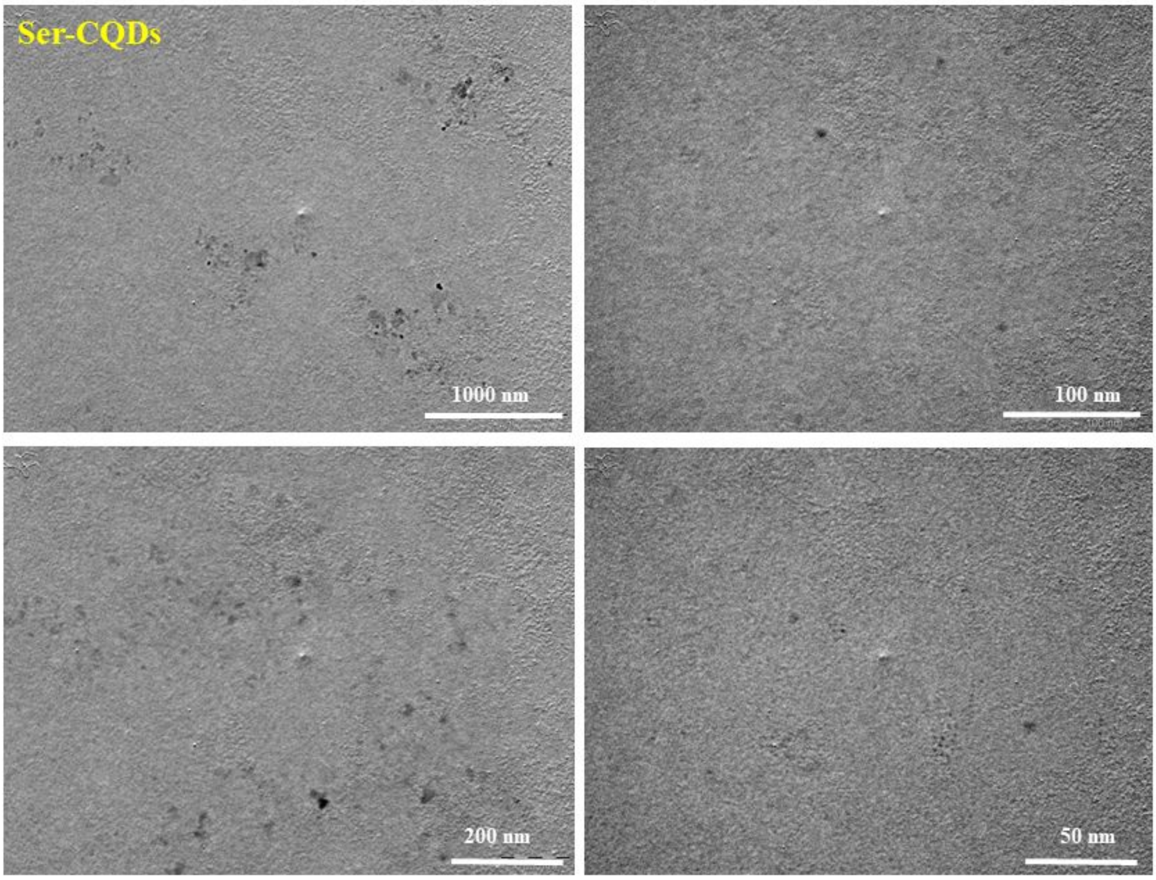

Figure S14: TEM micrograph of serine derived CQDs (Ser-CQDs) from serine molecules.

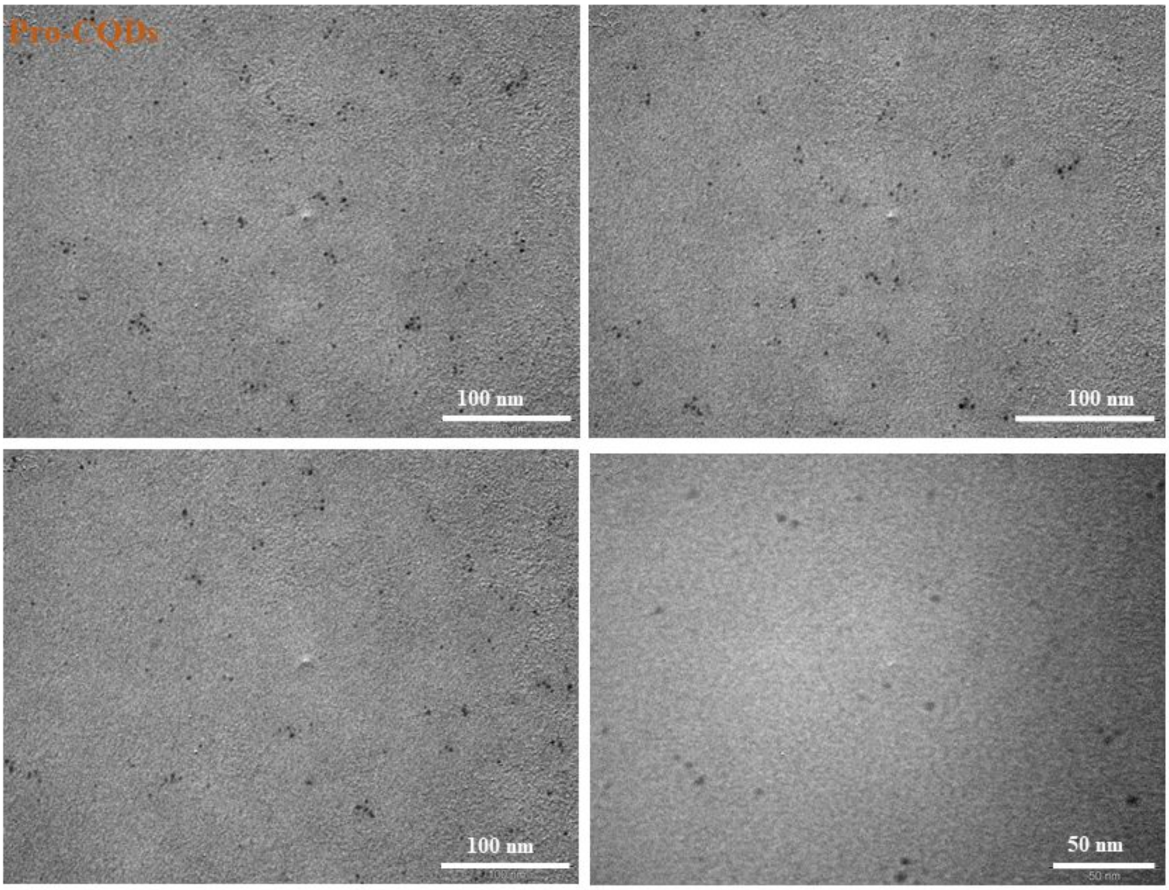

Figure S15: TEM micrograph of proline derived CQDs (Pro-CQDs) from proline molecules.

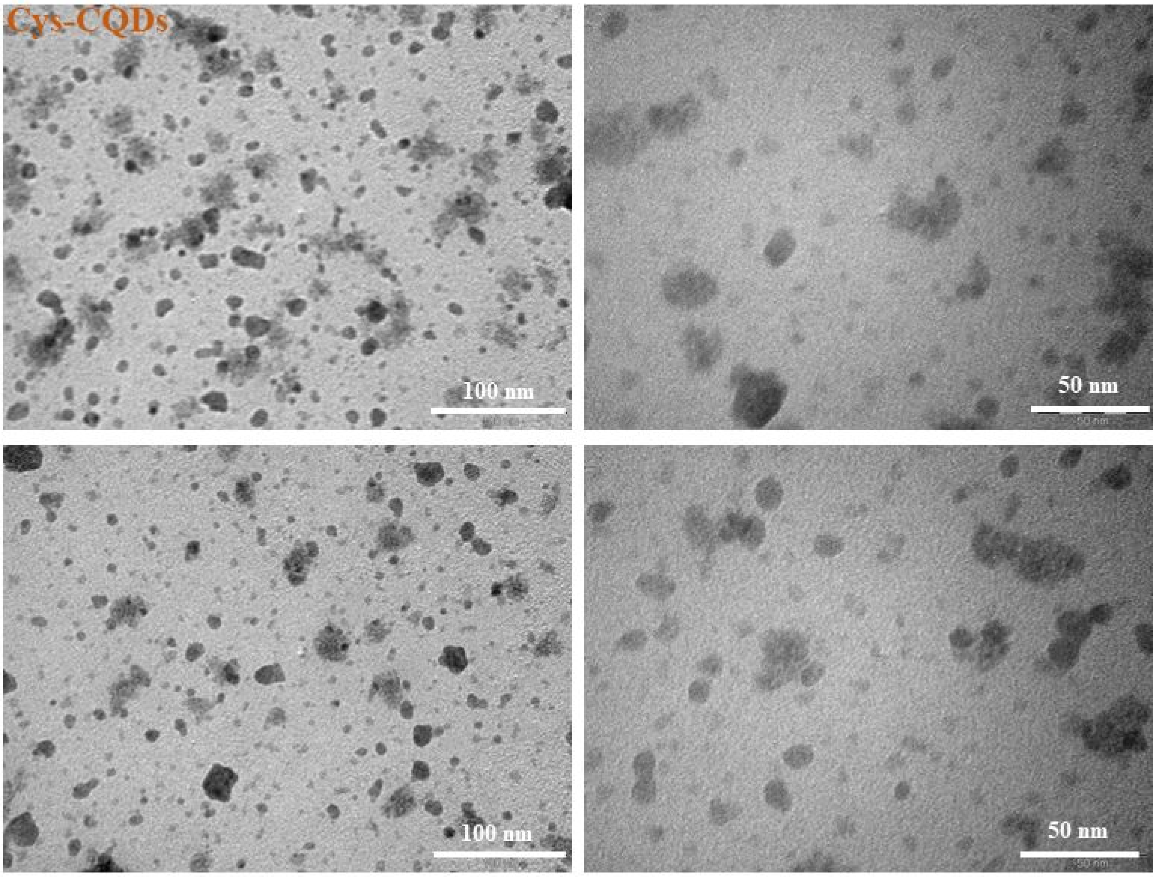

Figure S16: TEM micrograph of cysteine derived CQDs (Cys-CQDs) from cysteine molecules.

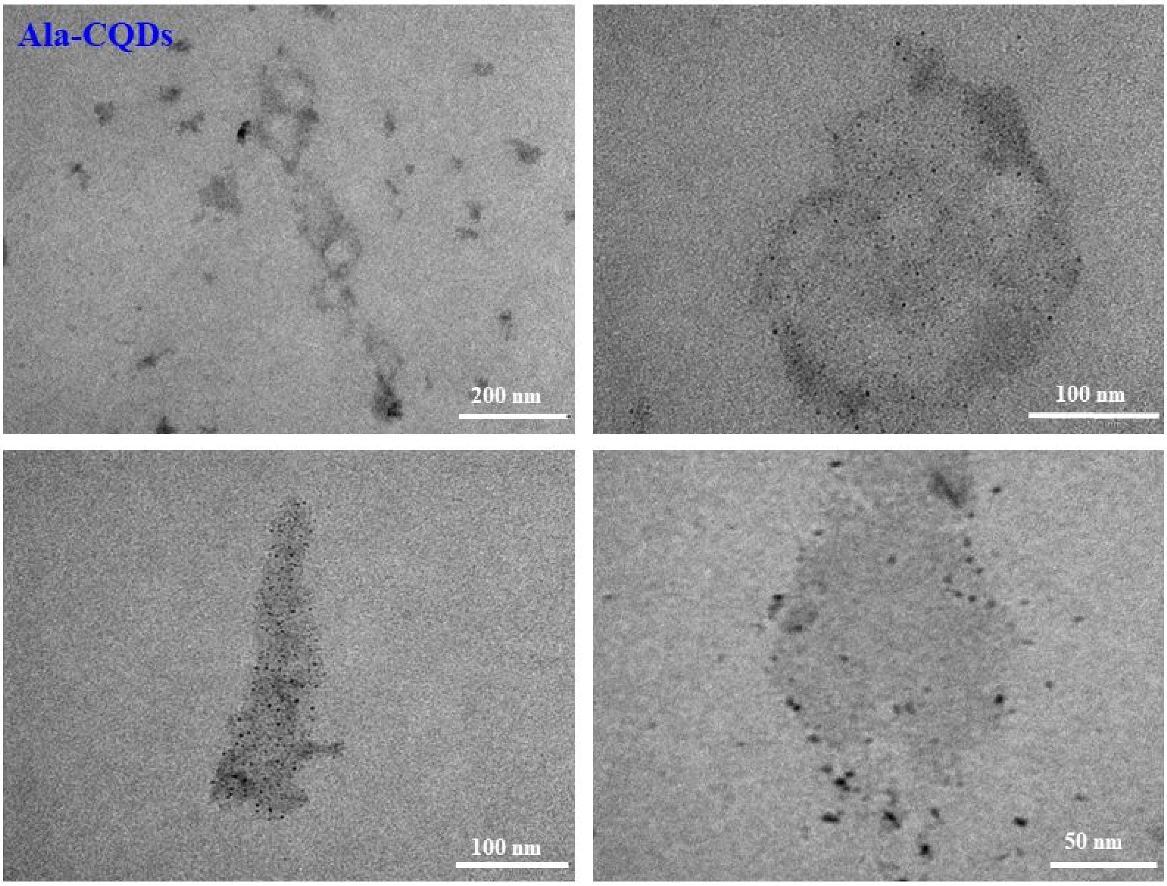

Figure S17: TEM micrograph of alanine derived CQDs (Ala-CQDs) from alanine molecules.

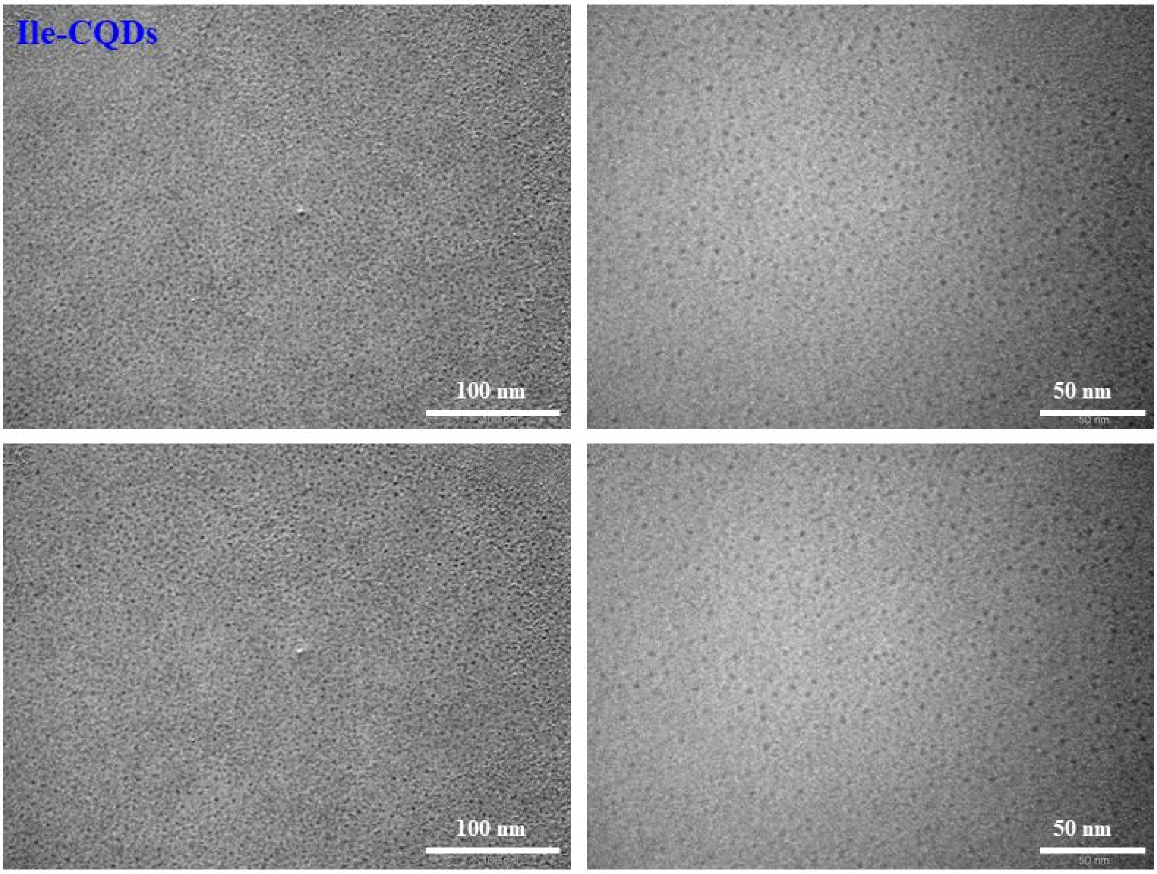

Figure S18: TEM micrograph of isoleucine derived CQDs (Ile-CQDs) from isoleucine molecules.

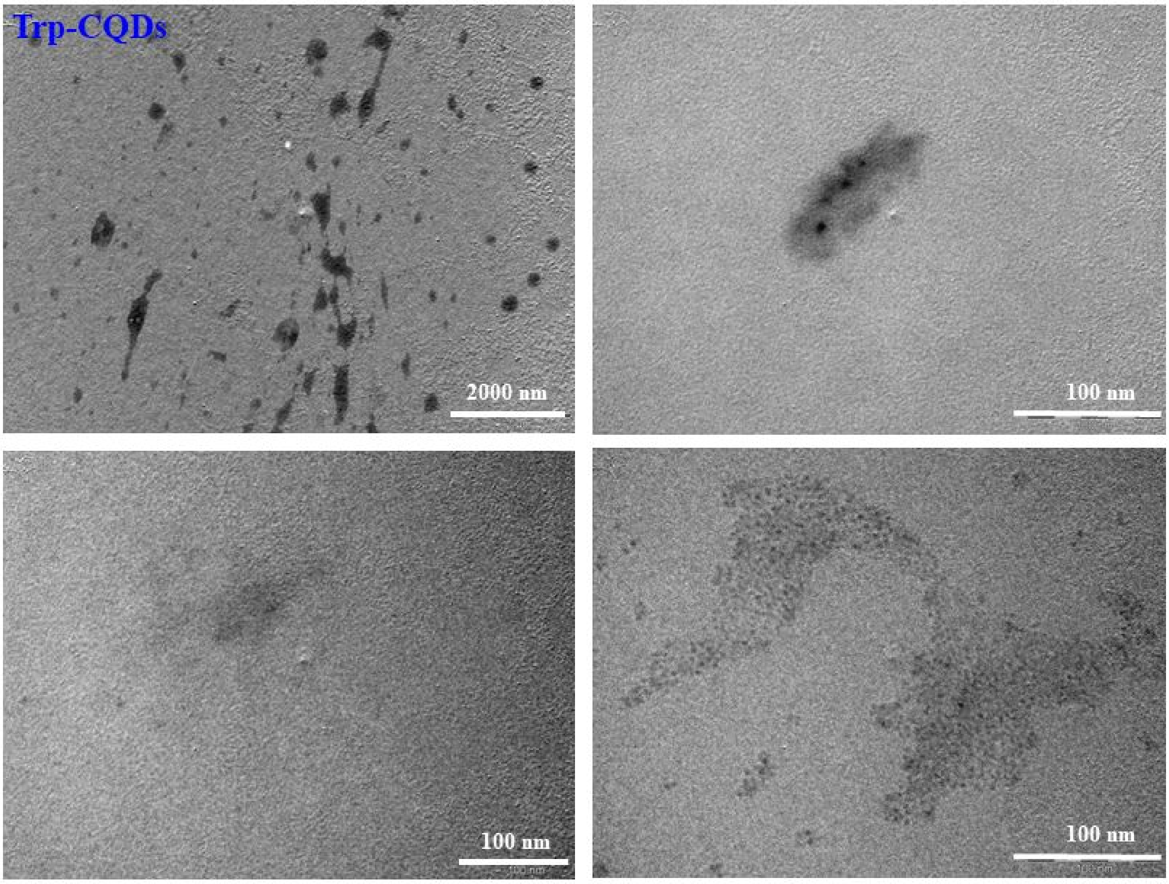

Figure S19: TEM micrograph of tryptophan derived CQDs (Trp-CQDs) from tryptophan molecules.

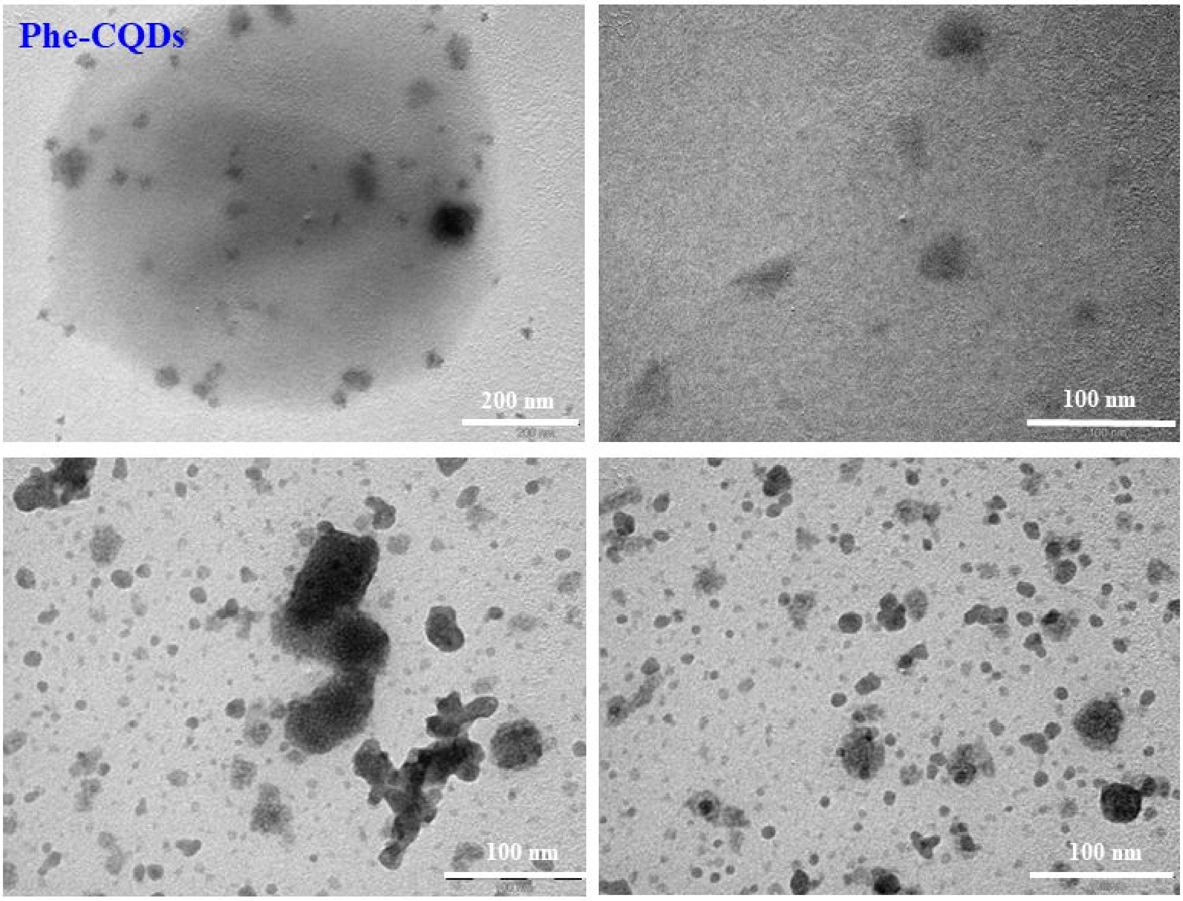

Figure S20: TEM micrograph of Phenyl alanine derived CQDs (Phe-CQDs) from phenylalanine molecules.

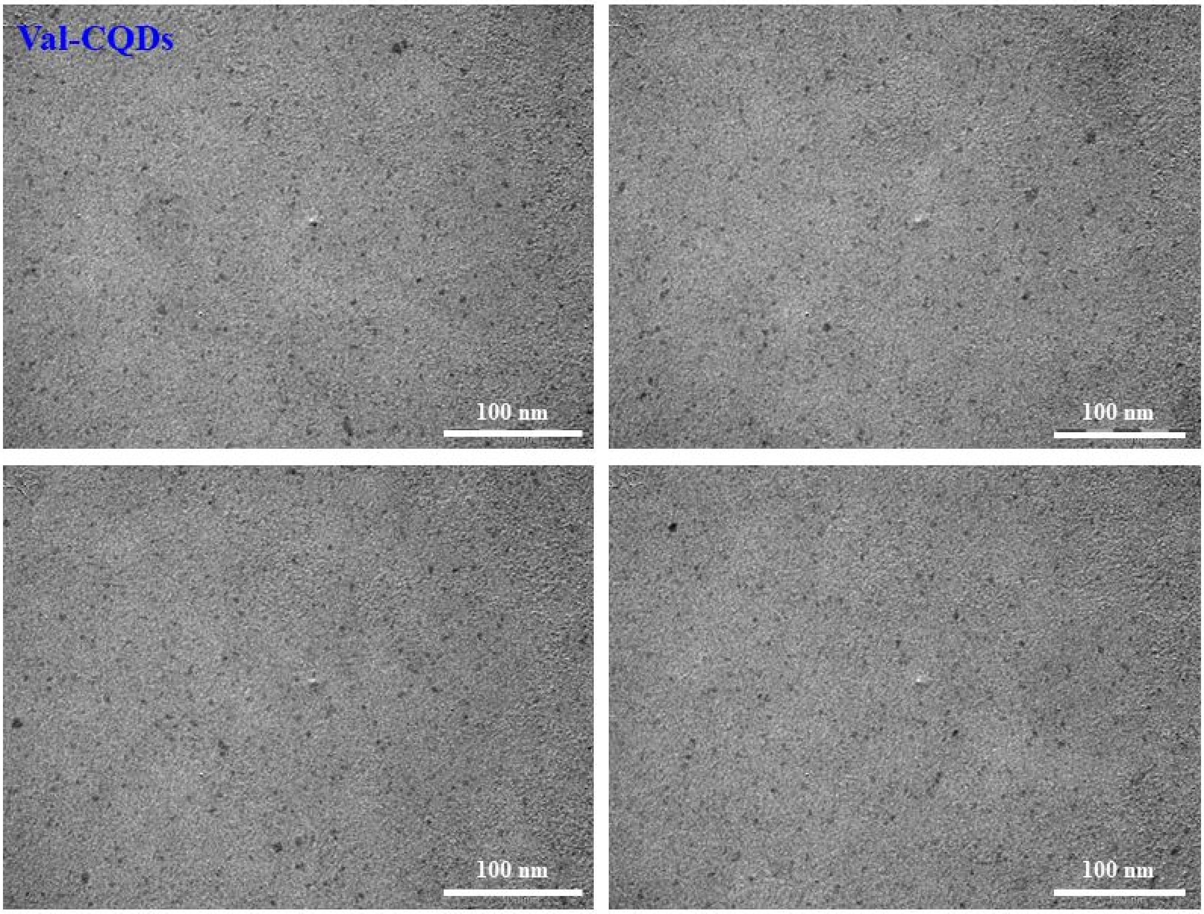

Figure S21: TEM micrograph of Valine derived CQDs (Val-CQDs) from valine molecules.

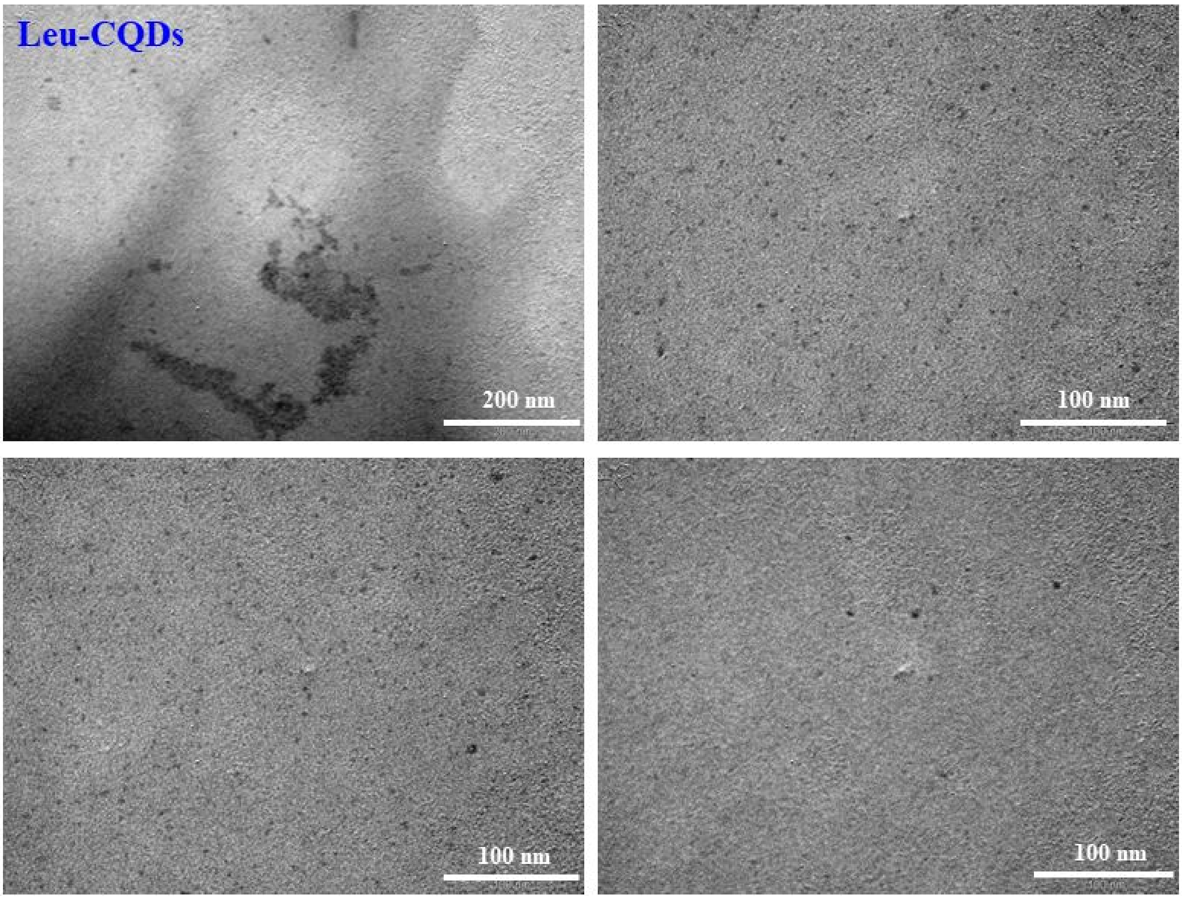

Figure S22: TEM micrograph of leucine derived CQDs (Leu-CQDs) from leucine molecules.

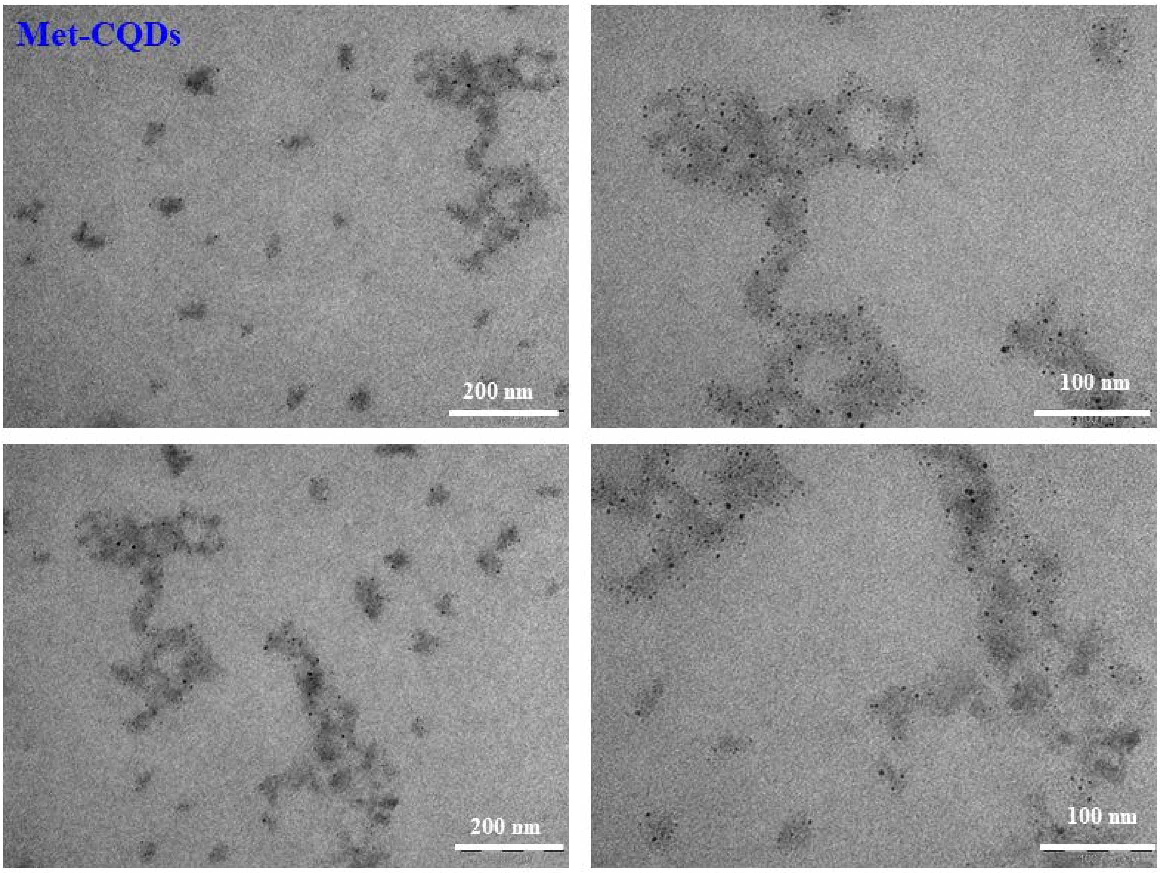

Figure S23: TEM micrograph of methionine derived CQDs (Met-CQDs) from methionine molecules.

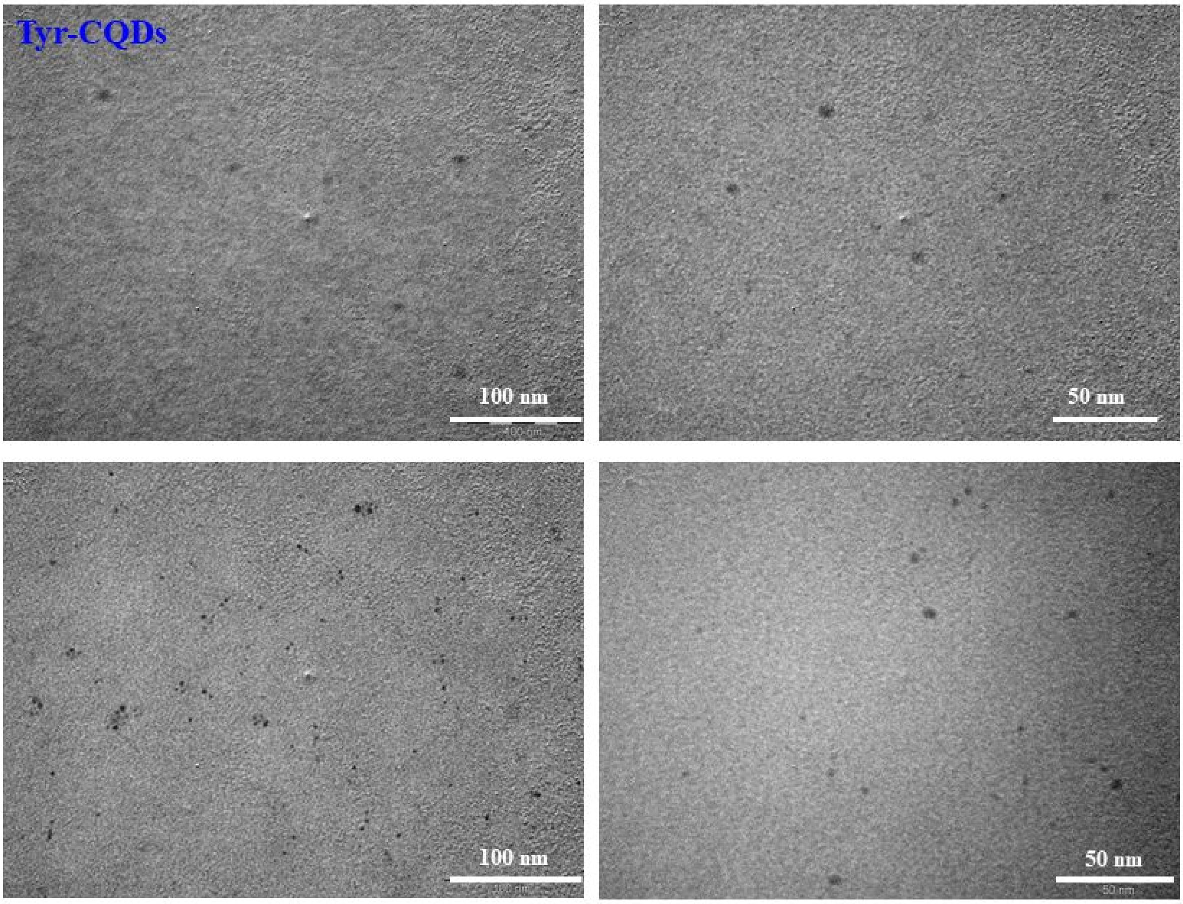

Figure S24: TEM micrograph of tyrosine derived CQDs (Tyr-CQDs) from tyrosine molecules.

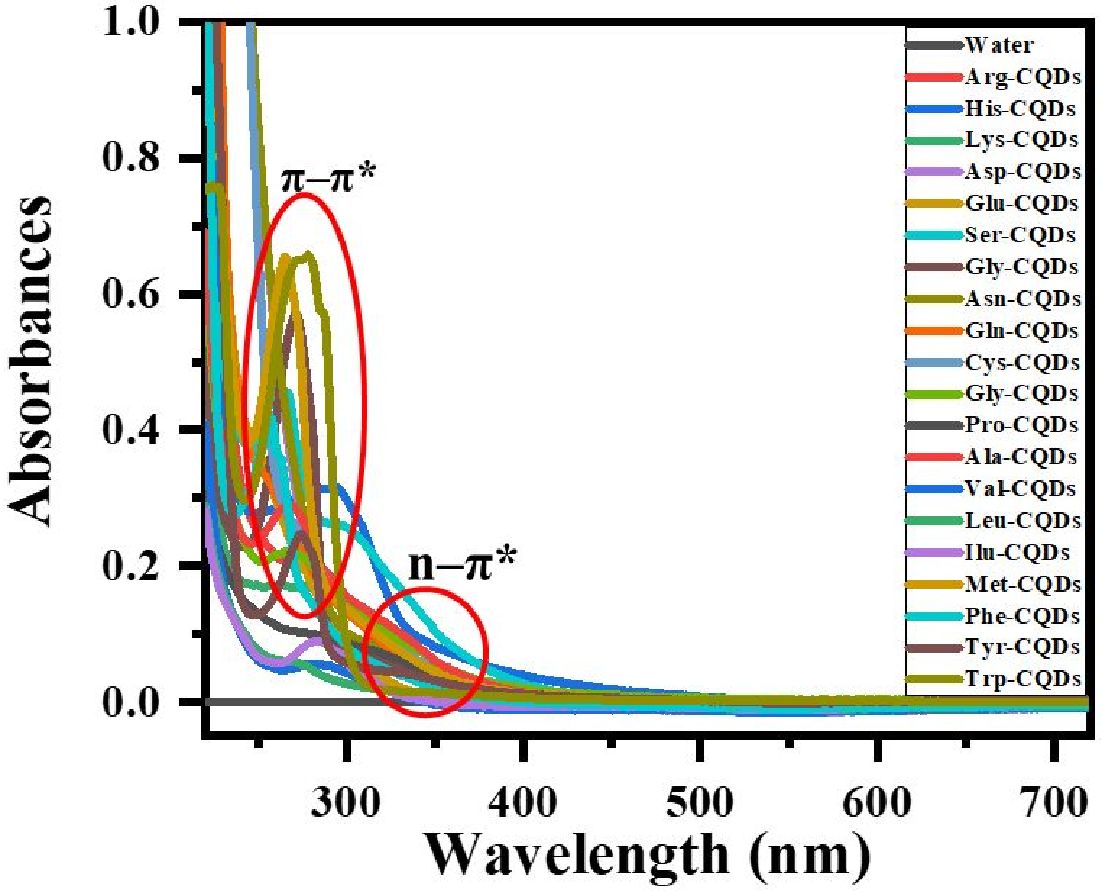

Figure S25. A comparison of the UV–vis spectra of each of the 20 different amino acid-derived CQDs with different amino acid functionalizations.

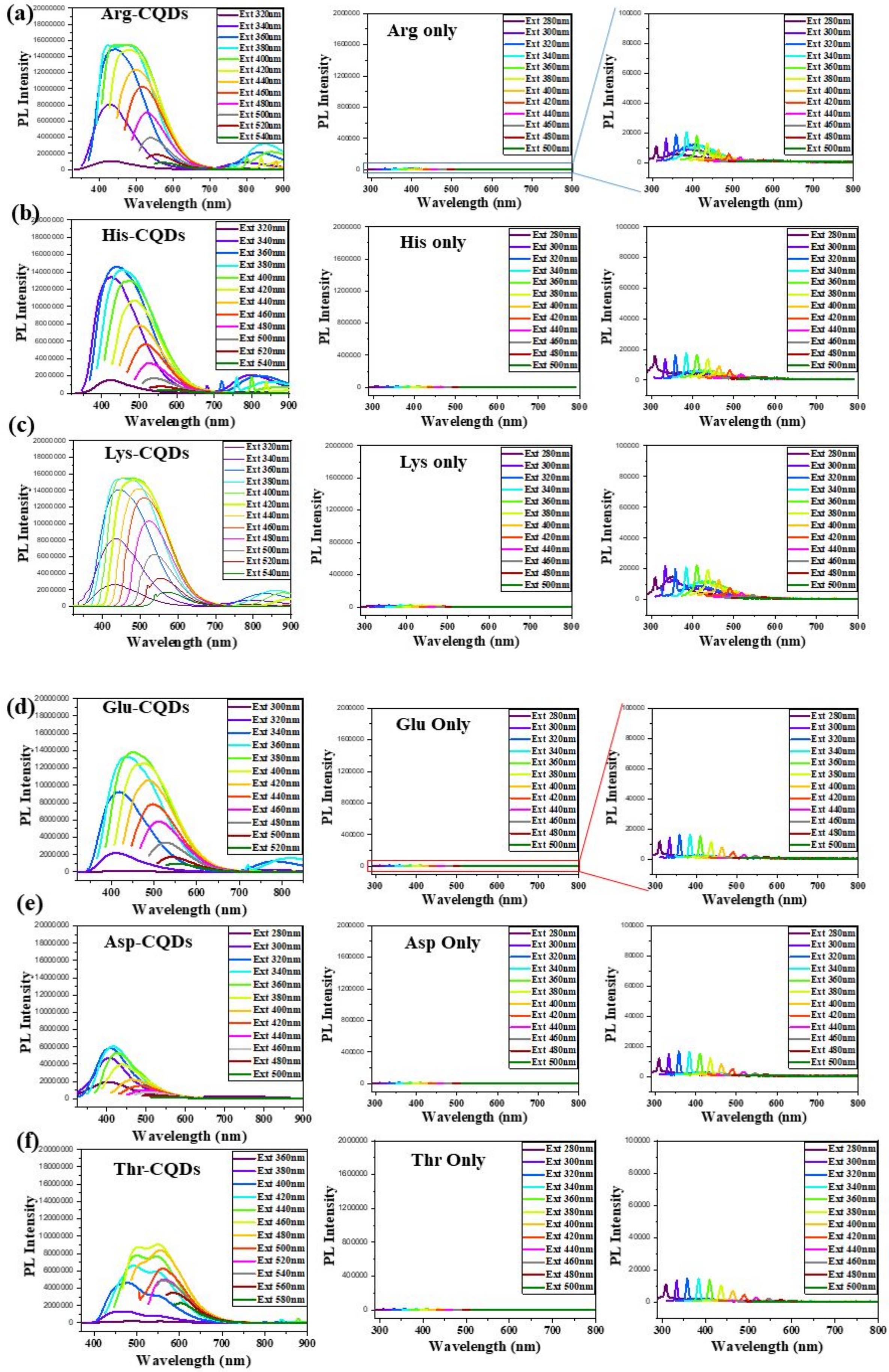
(a)
Arg-CQDs
Ext 320nm
Ext 340nm
Ext 360nm
Ext 380nm
Ext 400nm
Ext 420nm
Ext 440nm
Ext 460nm
Ext 480nm
Ext 500nm
Ext 520nm
Ext 540nm
PL Intensity
Wavelength (nm)
Arg only
Ext 280nm
Ext 300nm
Ext 320nm
Ext 340nm
Ext 360nm
Ext 380nm
Ext 400nm
Ext 420nm
Ext 440nm
Ext 460nm
Ext 480nm
Ext 500nm
(b)
His-CQDs
His only
(c)
Lys-CQDs
Lys only
(d)
Glu-CQDs
Ext 300nm
Glu Only
(e)
Asp-CQDs
Asp Only
(f)
Thr-CQDs
Ext 360nm
Ext 380nm
Ext 400nm
Ext 420nm
Ext 440nm
Ext 460nm
Ext 480nm
Ext 500nm
Ext 520nm
Ext 540nm
Ext 560nm
Ext 580nm
Thr Only

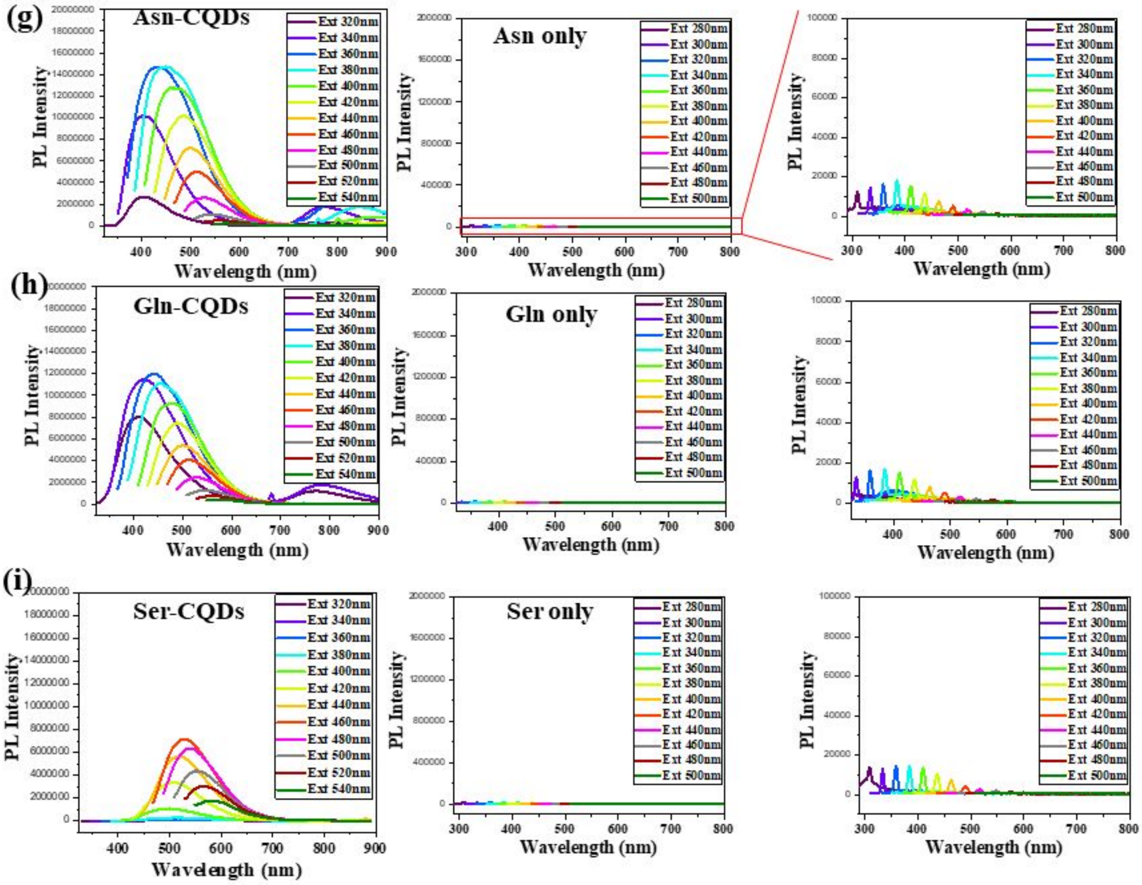
(g)
Asn-CQDs
Asn only
(h)
Gln-CQDs
Gln only
(i)
Ser-CQDs
Ser only
PL Intensity
Wavelength (nm)
Ext 280nm
Ext 300nm
Ext 320nm
Ext 340nm
Ext 360nm
Ext 380nm
Ext 400nm
Ext 420nm
Ext 440nm
Ext 460nm
Ext 480nm
Ext 500nm
Ext 520nm
Ext 540nm

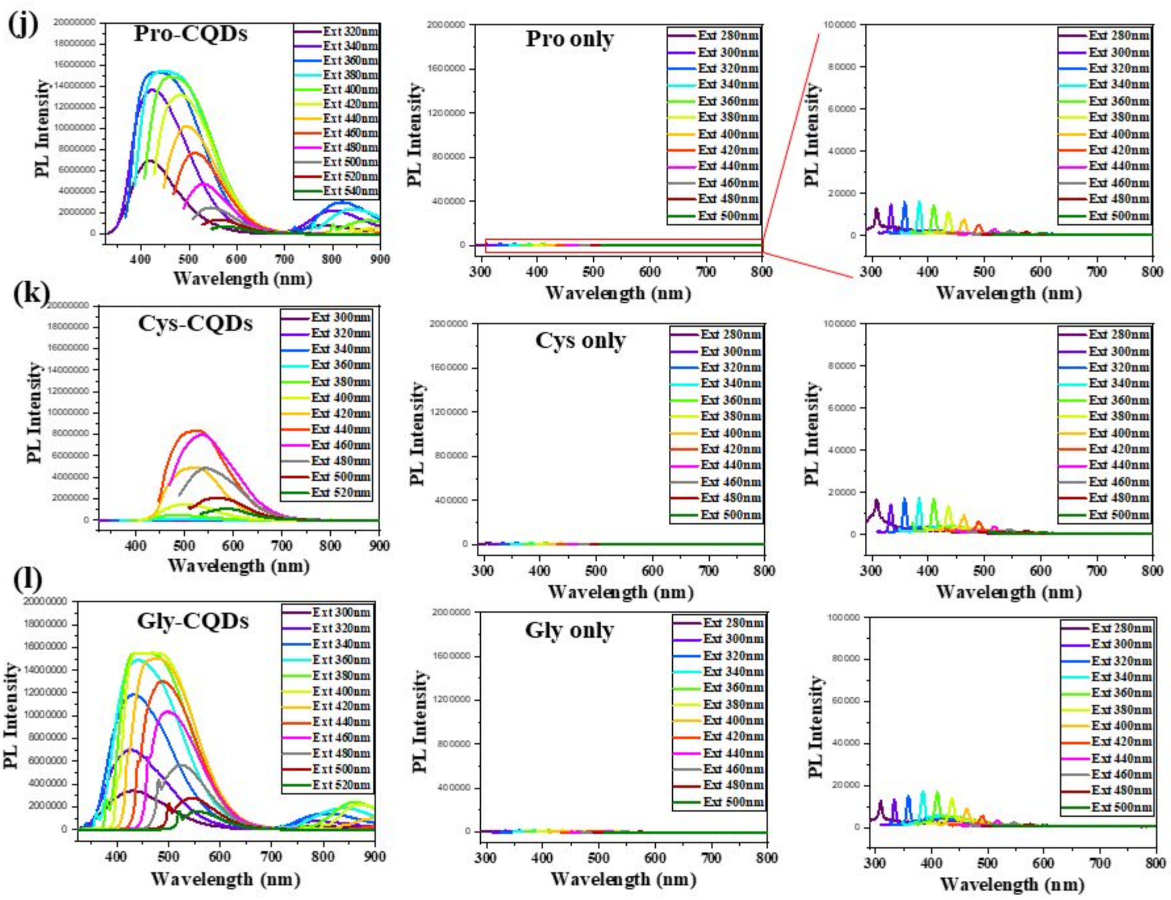
(j)
Pro-CQDs
Ext 320nm
Ext 340nm
Ext 360nm
Ext 380nm
Ext 400nm
Ext 420nm
Ext 440nm
Ext 460nm
Ext 480nm
Ext 500nm
Ext 520nm
Ext 540nm
PL Intensity
Wavelength (nm)
Pro only
Ext 280nm
Ext 300nm
Ext 320nm
Ext 340nm
Ext 360nm
Ext 380nm
Ext 400nm
Ext 420nm
Ext 440nm
Ext 460nm
Ext 480nm
Ext 500nm
(k)
Cys-CQDs
Ext 300nm
Ext 320nm
Ext 340nm
Ext 360nm
Ext 380nm
Ext 400nm
Ext 420nm
Ext 440nm
Ext 460nm
Ext 480nm
Ext 500nm
Ext 520nm
Cys only
(l)
Gly-CQDs
Gly only

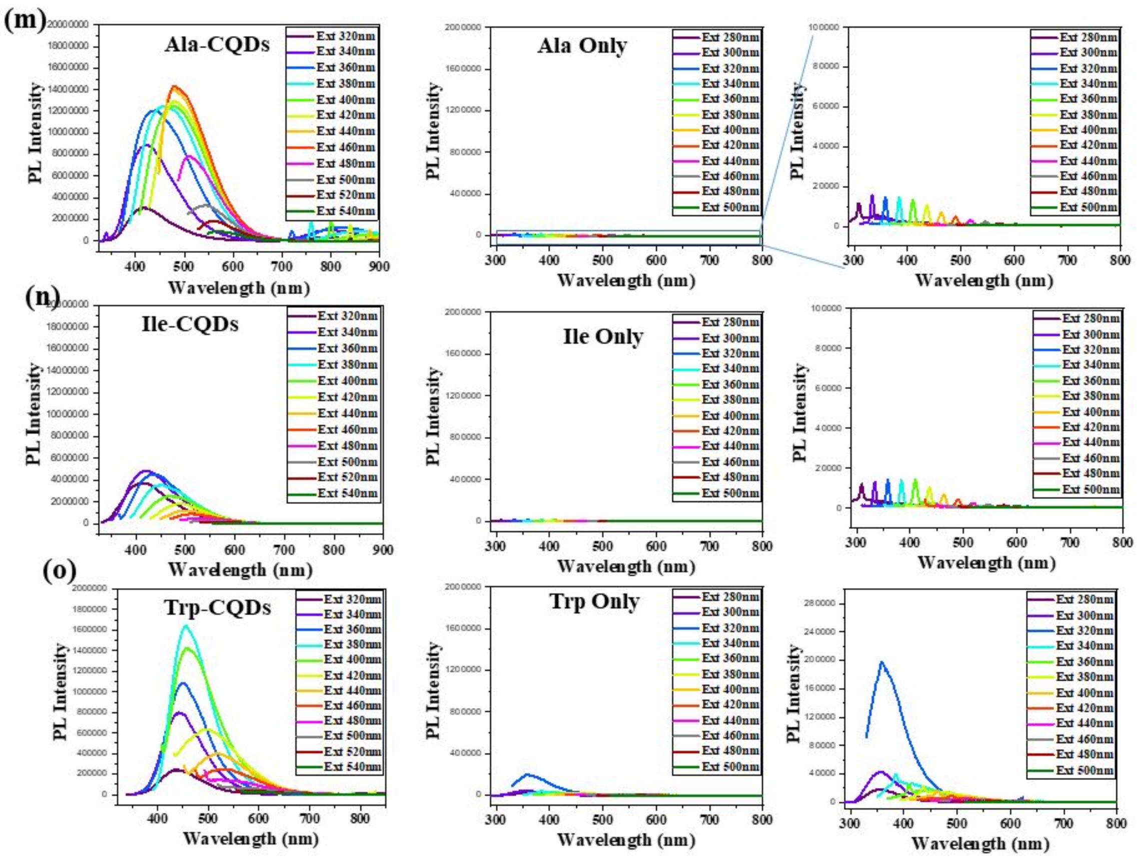
(m)
Ala-CQDs
Ala Only
PL Intensity
Wavelength (nm)
Ext 320nm
Ext 340nm
Ext 360nm
Ext 380nm
Ext 400nm
Ext 420nm
Ext 440nm
Ext 460nm
Ext 480nm
Ext 500nm
Ext 520nm
Ext 540nm
Ext 280nm
Ext 300nm
(n)
Ile-CQDs
Ile Only
(o)
Trp-CQDs
Trp Only

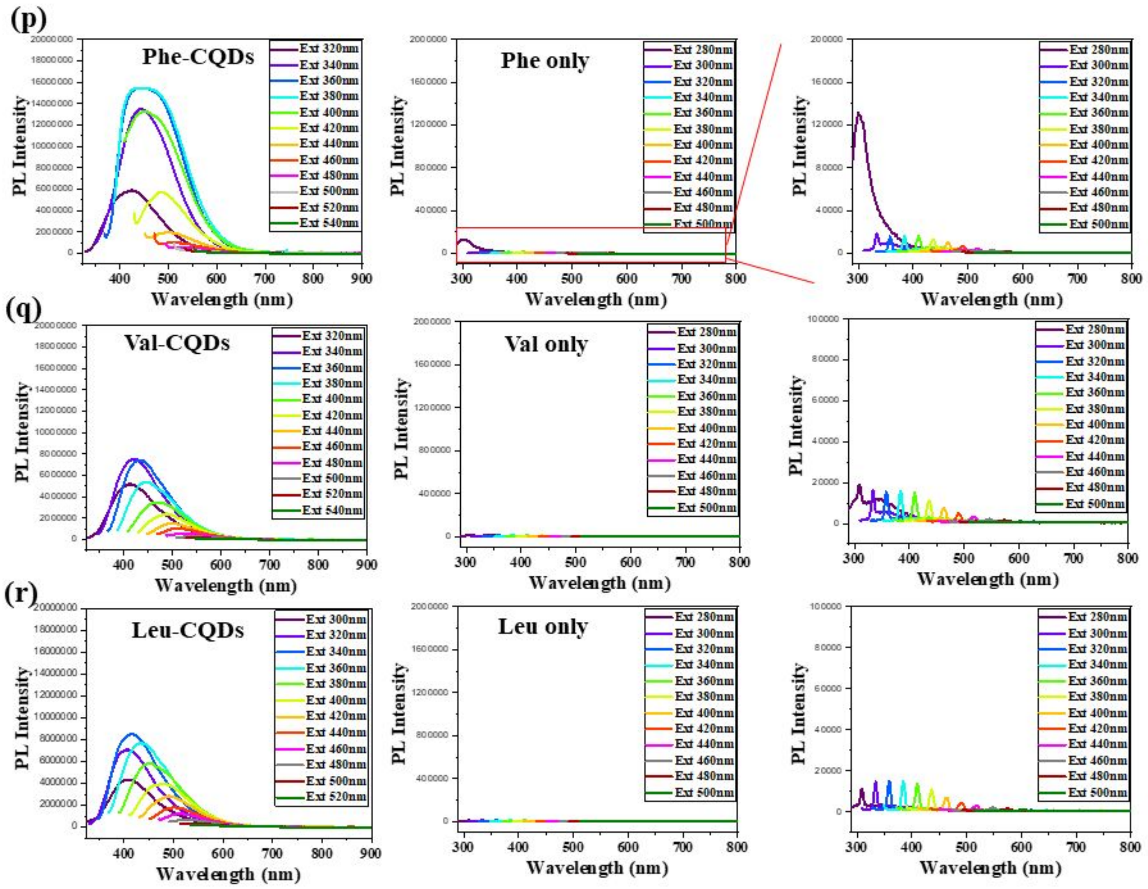
(p)
Phe-CQDs
Phe only
PL Intensity
Wavelength (nm)
Ext 320nm
Ext 340nm
Ext 360nm
Ext 380nm
Ext 400nm
Ext 420nm
Ext 440nm
Ext 460nm
Ext 480nm
Ext 500nm
Ext 520nm
Ext 540nm
Ext 280nm
Ext 300nm
(q)
Val-CQDs
Val only
(r)
Leu-CQDs
Leu only

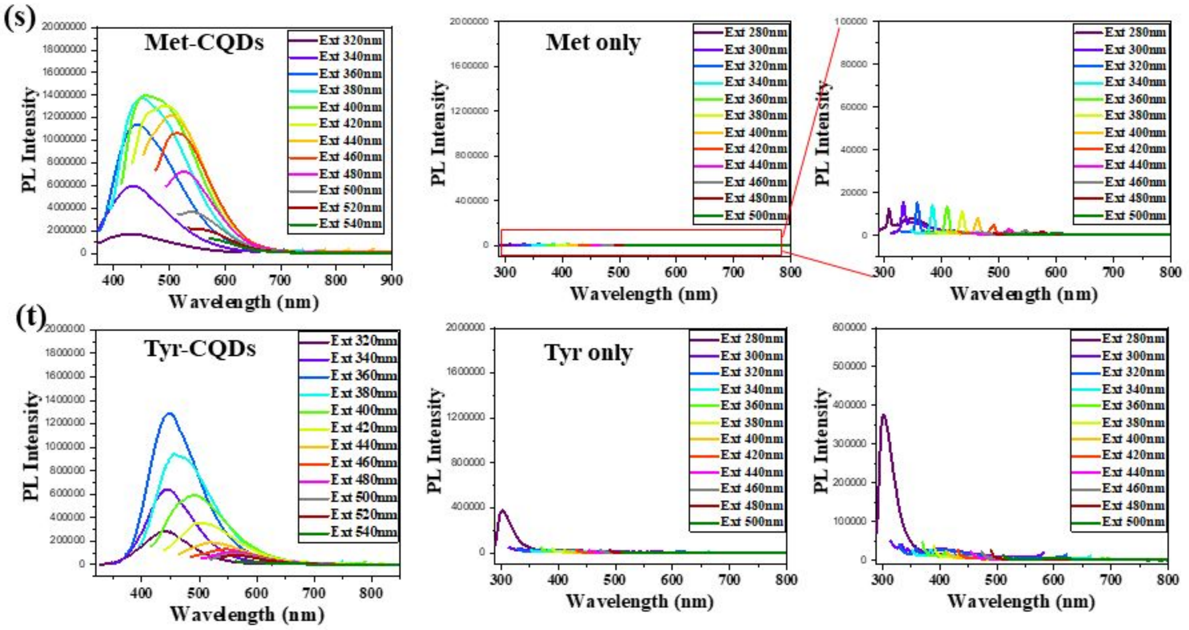

Figure S26: A comparison of fluorescence analysis graphs for all the AAs derived CQDs and pristine solutions of all amino acids: (a) Arg-CQDs and pristine Arg, (b) His-CQDs and pristine histidine, (c) Lys-CQDs and pristine aqueous solutions of lysine, (d) Glu-CQDs and a pristine aqueous solution of glutamic acid, (e) Asp-CQDs and a pristine aqueous solution of aspartic acid, (f) Thr-CQDs and a pristine aqueous solution of threonine, (g) Asn-CQDs and a pristine aqueous solution of asparagine, (h) Gln-CQDs and a pristine aqueous solution of glutamine, (i) Ser-CQDs and a pristine aqueous solution of serine, (j) Pro-CQDs and pristine aqueous solution of proline, (k) Cys-CQDs and a pristine aqueous solution of cysteine, (l) Gly-CQDs and a pristine aqueous solution of glycine, (m) Ala-CQDs and a pristine aqueous solution of alanine, (n) Ile-CQDs and pristine aqueous solution of isoleucine, (o) Trp-CQDs and a pristine aqueous solution of tryptophan, (p) Phe-CQDs and a pristine aqueous solution of phenylalanine, (q) Val-CQDs and pristine aqueous solution of valine, Figure S24r for Leu-CQDs and a pristine aqueous solution of leucine, (s) Met-CQDs and a pristine aqueous solution of methionine, and (t) Tyr-CQDs and a pristine aqueous solution of tyrosine.

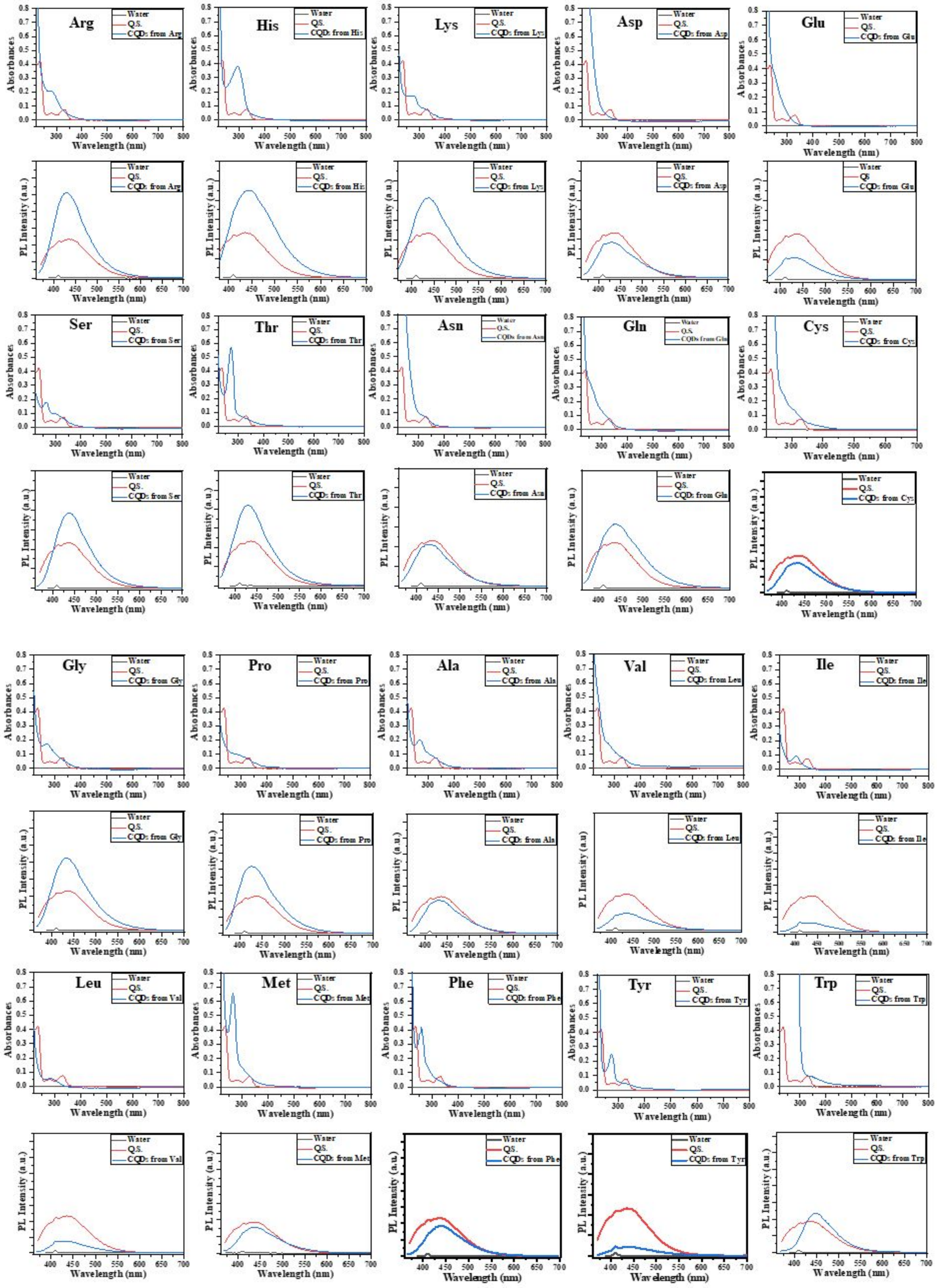

Figure S27: The quantum yeild of all 20 amino acid dervied CQDs at 360 nm and campared the QS.

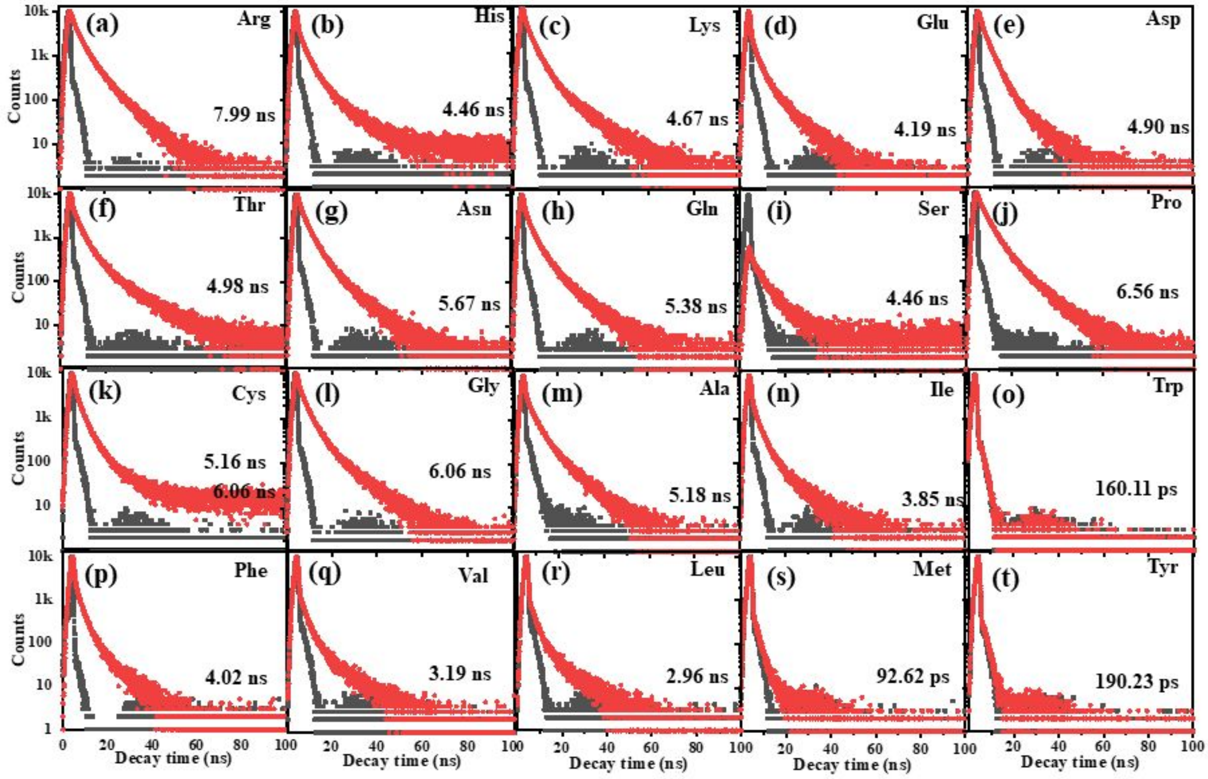

Figure S28. Time-resolved Photoluminescence decay time spectra of all 20 AA-CQDs. (a) Arg-CQDs, (b) His-CQDs, (c) Lys-CQDs, (d) Glu-CQDs, (e) Asp-CQDs, (f) Thr-CQDs, (g) Asn-CQDs, (h) Gln-CQDs, (i) Ser-CQDs, (j) Pro-CQDs, (k) Cys-CQDs, (l) Gly-CQDs, (m) Ala-CQDs, (n) Ile-CQDs, (o) Trp-CQDs, (p) Phe-CQDs, (q) Val-CQDs, (r) Leu-CQDs, (s) Met-CQDs, and (t) Tyr-CQDs. Y-axes are logarithmic.

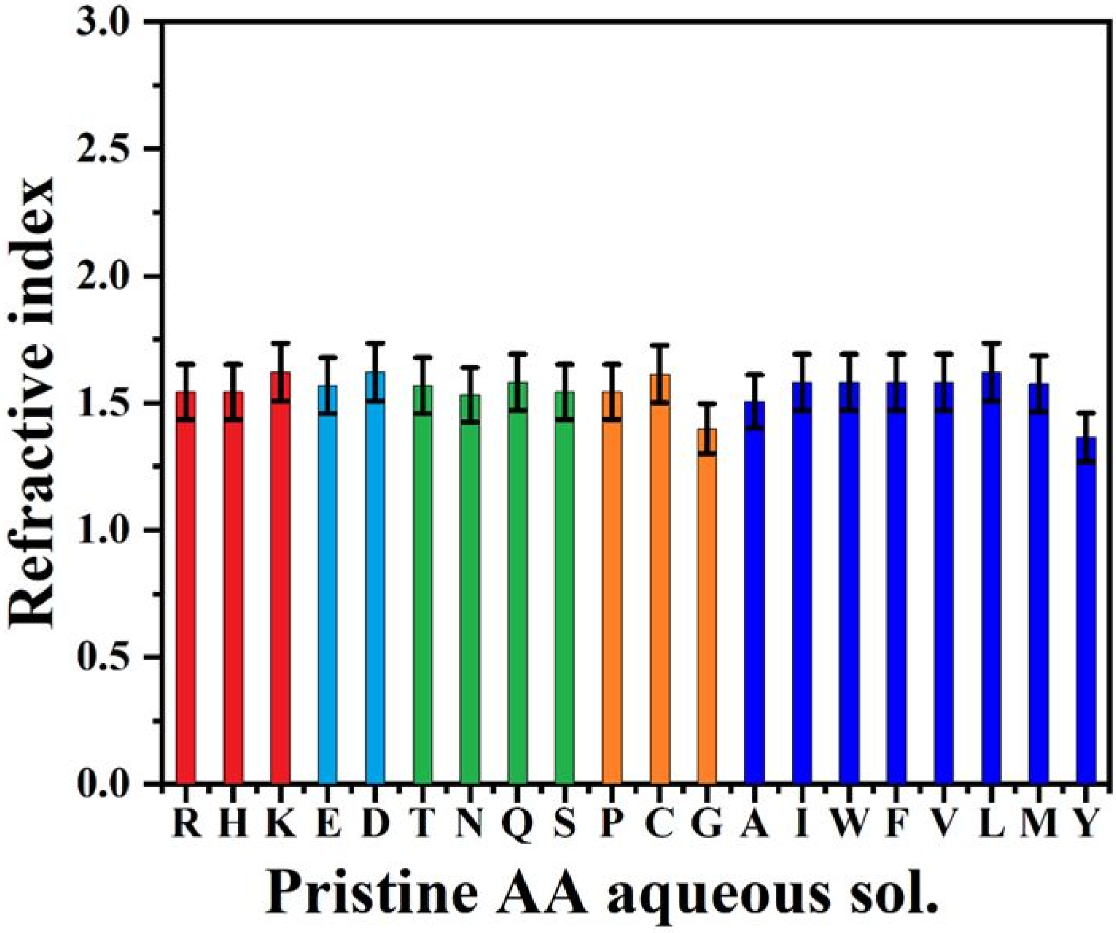

Figure: S29. A Graphical representation of RI of all amino acid by measuring the RI by refractometer at 589 nm. Red: positively charged, cyan: negatively charged, green: uncharged side chain, orange: special cases, blue: hydrophobic side chain.

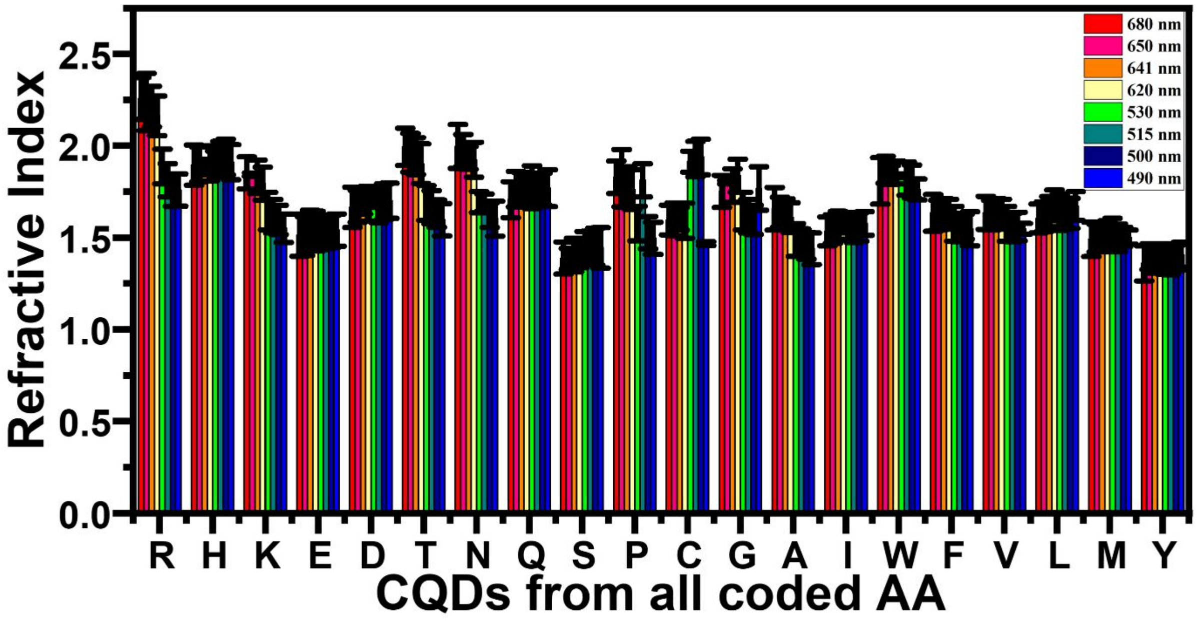

Figure: S30. A Graphical representation of RI of amino acid derived CQDs by measuring the RI of CQDs using hologram and microchannel method at different wavelengths (680nm, 650nm, 641nm, 620nm, 530nm, 515nm, 500nm, 490nm).

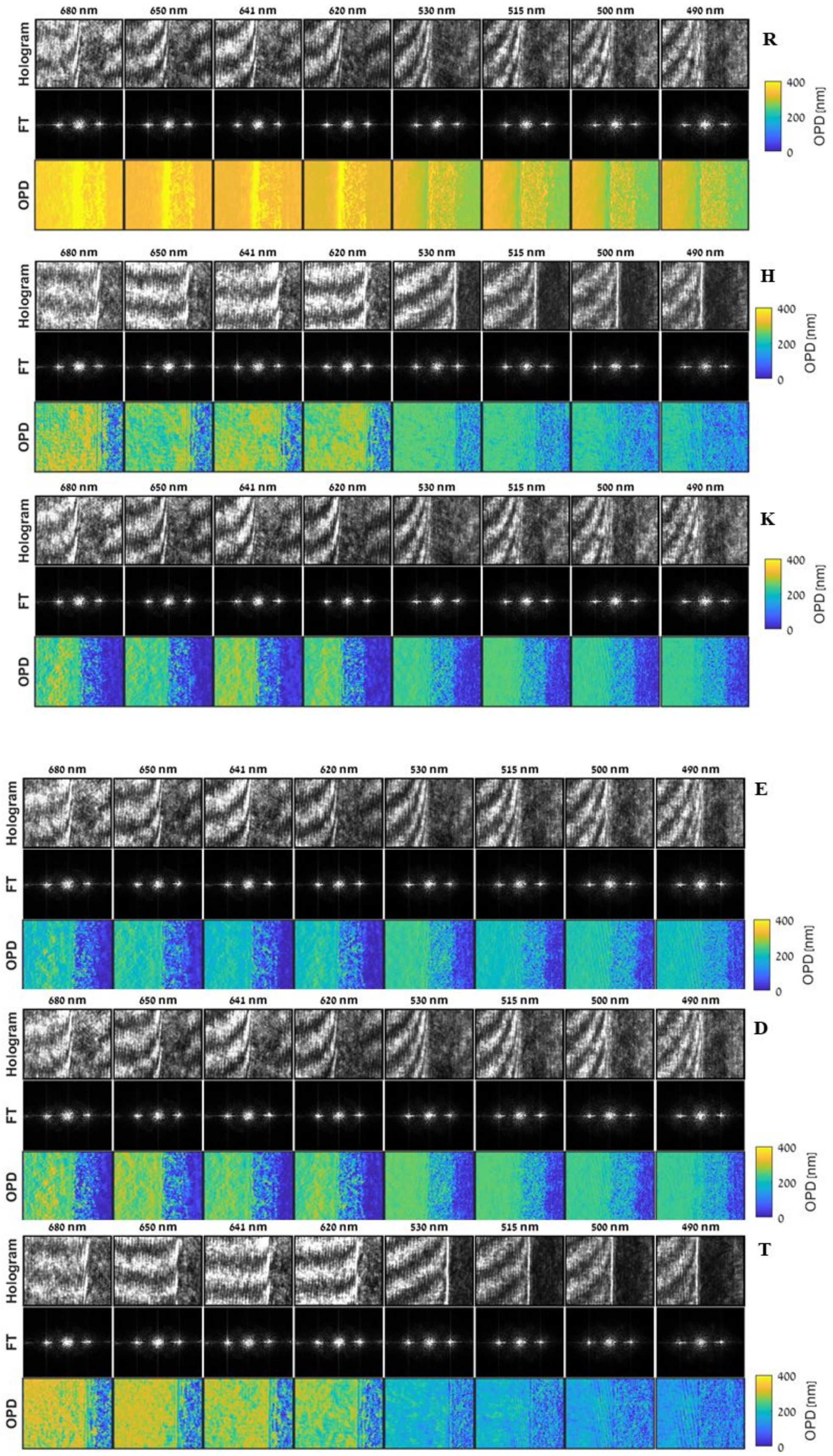

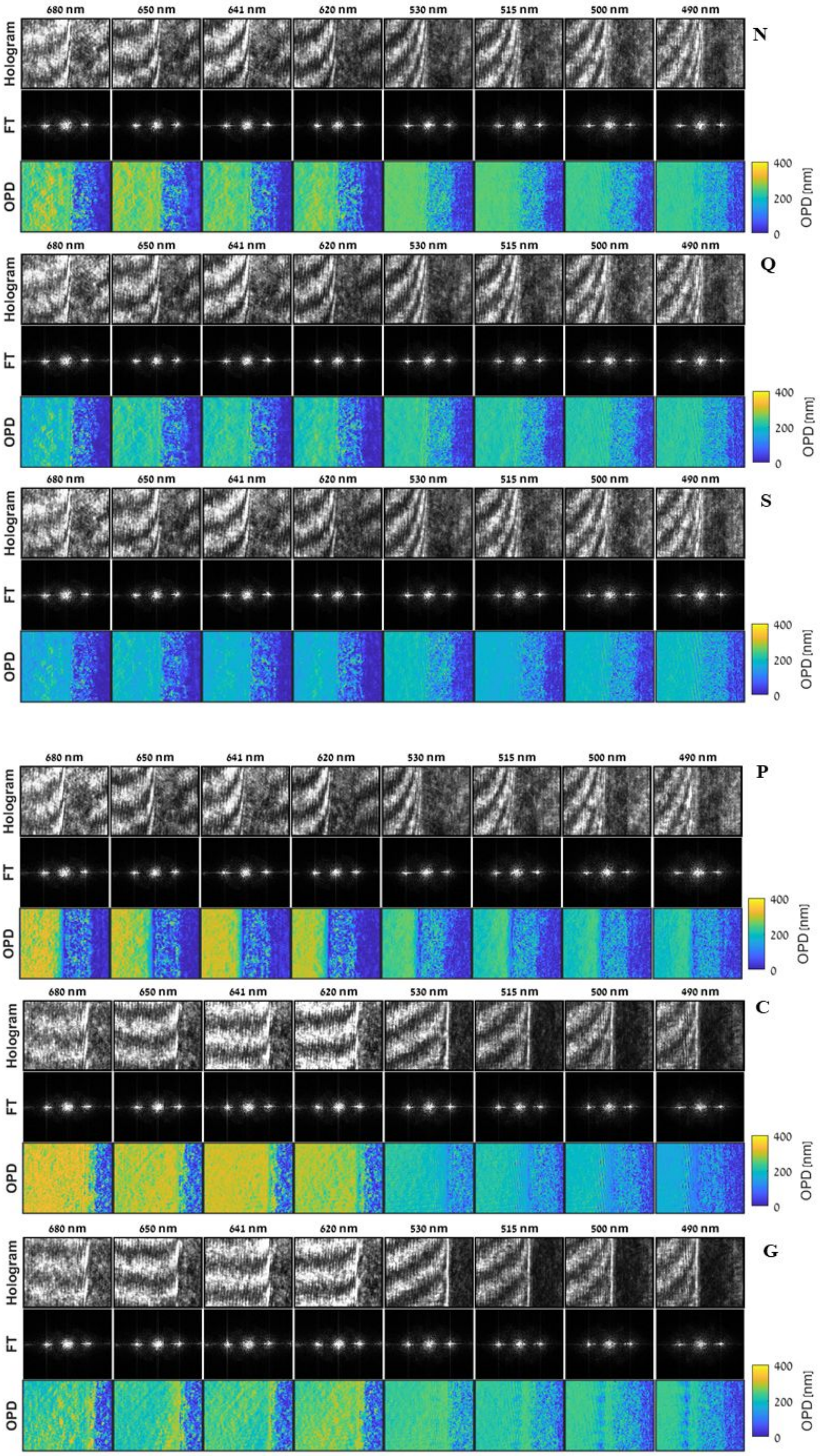
680 nm
650 nm
641 nm
620 nm
530 nm
515 nm
500 nm
490 nm
Hologram
FT
OPD
OPD [nm]
400
200
0
N
Q
S
P
C
G

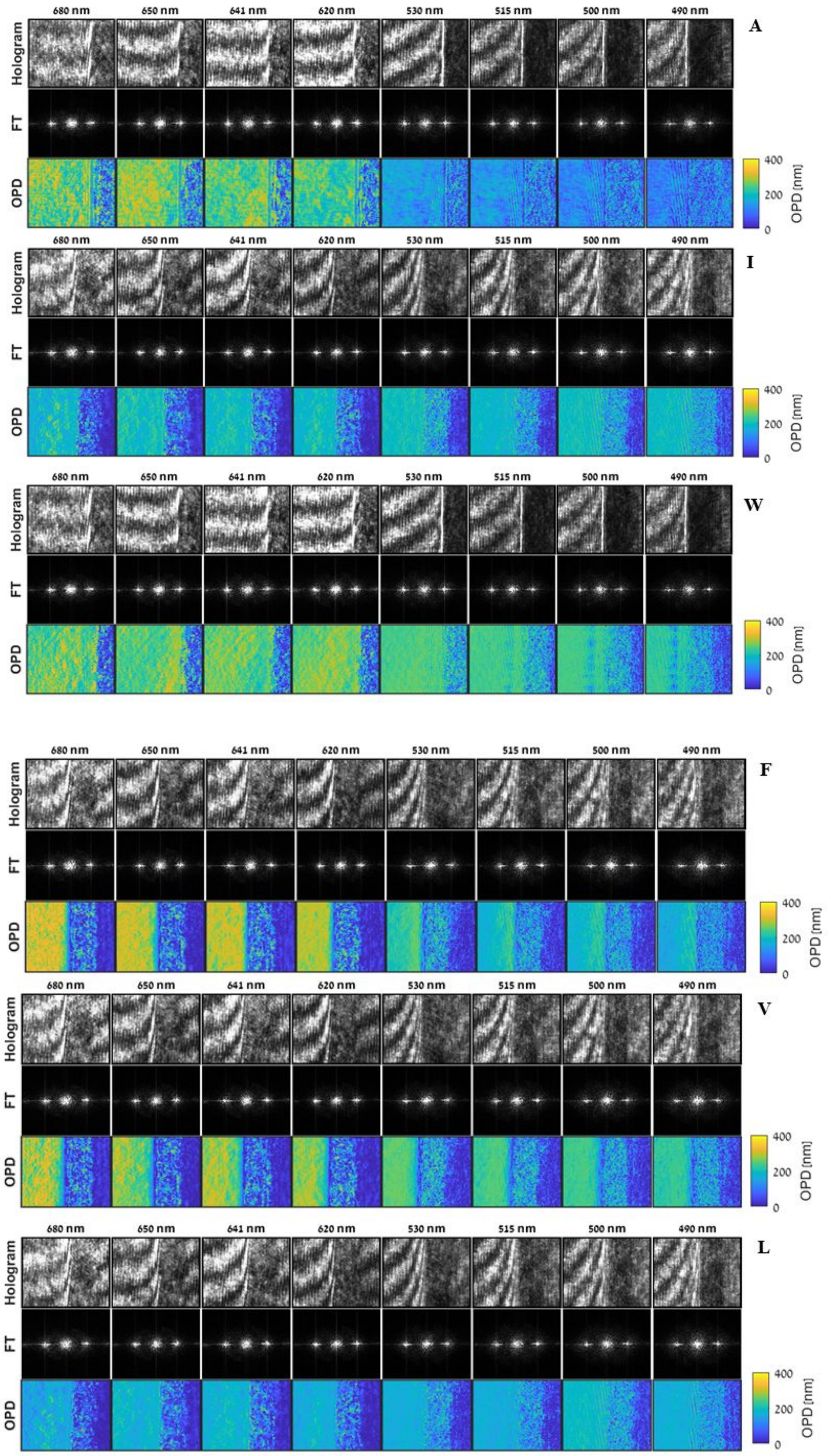

680 nm
650 nm
641 nm
620 nm
530 nm
515 nm
500 nm
490 nm
Hologram
FT
OPD
OPD [nm]
400
200
0
A
I
W
F
V
L

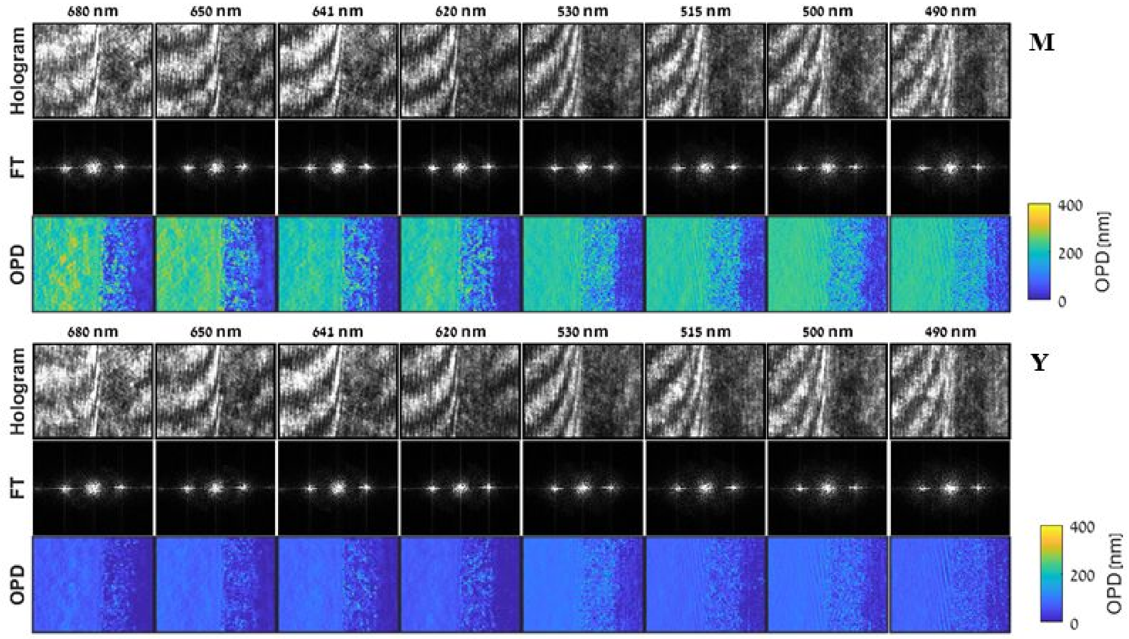

Figure: S31. A Graphical representation of RI of amino acid derived CQDs and measuring by IPM technique at different wavelengths (680nm, 650nm, 641nm, 620nm, 530nm, 515nm, 500nm, 490nm). (a) Arg-CQDs, (b) His-CQDs, (c) Lys-CQDs, (d) Glu-CQDs, (e) Asp-CQDs, (f) Thr-CQDs, (g) Asn-CQDs, (h) Gln-CQDs, (i) Ser-CQDs, (j) Pro-CQDs, (k) Cys-CQDs, (l) Gly-CQDs, (m) Ala-CQDs, (n) Ile-CQDs, (o) Trp-CQDs, (p) Phe-CQDs, (q) Val-CQDs, (r) Leu-CQDs, (s) Met-CQDs, and (t) Tyr-CQDs.

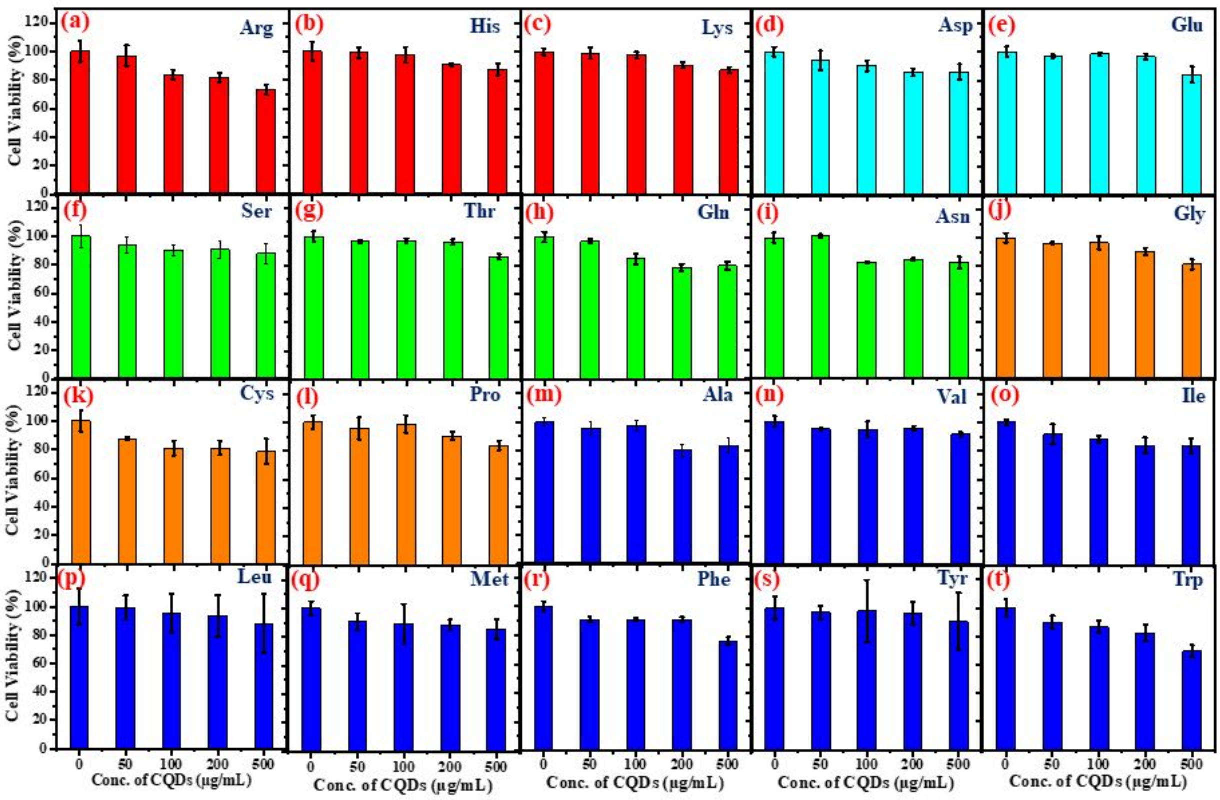

Figure: S32. Cell viability measurements of all 20 AA-CQDs by MTT assay on Hela cell line. Red: positively charged, cyan: negatively charged, green: uncharged side chain, orange: special cases, blue: hydrophobic side chain. (a) Arg-CQDs, (b) His-CQDs, (c) Lys-CQDs, (d) Glu-CQDs, (e) Asp-CQDs, (f) Thr-CQDs, (g) Asn-CQDs, (h) Gln-CQDs, (i) Ser-CQDs, (j) Pro-CQDs, (k) Cys-CQDs, (l) Gly-CQDs, (m) Ala-CQDs, (n) Ile-CQDs, (o) Trp-CQDs, (p) Phe-CQDs, (q) Val-CQDs, (r) Leu-CQDs, (s) Met-CQDs, and (t) Tyr-CQDs. The Y-axis is cell viability and X-axis is concentration of AA-CQDs.

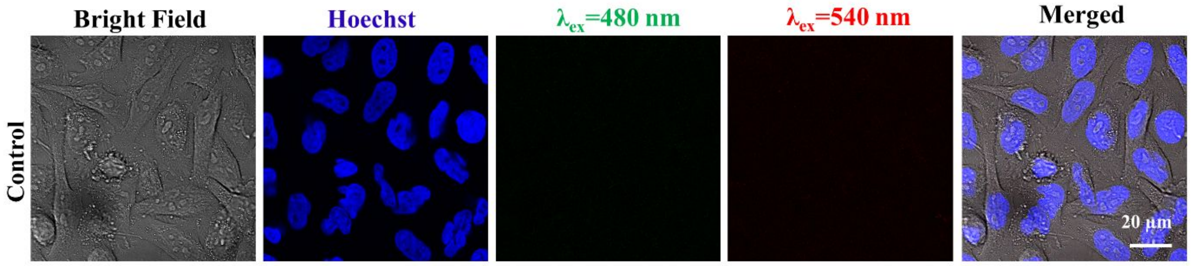

Figure: S33. Live cell imaging of Hela cell without any treatment with CQDs (as a control) using four fluorescence channels: bright field images, Hoechst (dye staining live cell nucleus), excitation at 480 nm. Scale bar: 20 µm.
